# A spatiotemporal transcriptomic atlas of mouse placentation

**DOI:** 10.1038/s41421-024-00740-6

**Published:** 2024-10-22

**Authors:** Yanting Wu, Kaizhen Su, Ying Zhang, Langchao Liang, Fei Wang, Siyue Chen, Ling Gao, Qiutong Zheng, Cheng Li, Yunfei Su, Yiting Mao, Simeng Zhu, Chaochao Chai, Qing Lan, Man Zhai, Xin Jin, Jinglan Zhang, Xun Xu, Yu Zhang, Ya Gao, Hefeng Huang

**Affiliations:** 1 Obstetrics and Gynecology Hospital, Institute of Reproduction and Development, Fudan University, Shanghai, China; 2 Key Laboratory of Reproductive Genetics (Ministry of Education), Zhejiang University School of Medicine, Hangzhou, Zhejiang China; 3 Research Units of Embryo Original Diseases, Chinese Academy of Medical Sciences (No. 2019RU056), Shanghai, China; 4 Shanghai Key Laboratory of Reproduction and Development, Shanghai, China; 5 Department of Reproductive Endocrinology, Women’s Hospital, Zhejiang University School of Medicine, Hangzhou, Zhejiang China; 6 International Peace Maternity and Child Health Hospital, School of Medicine, Shanghai Jiao Tong University, Shanghai, China; 7 BGI Research, Shenzhen, Guangdong China; 8 Shanxi Medical University - BGI Collaborative Center for Future Medicine, Shanxi Medical University, Taiyuan, Shanxi China; 9 BGI Research, Qingdao, Shandong China; 10 College of Life Sciences, University of Chinese Academy of Sciences, Beijing, China; 11 Department of Cardiology, Sixth People’s Hospital, Shanghai Jiao Tong University School of Medicine, Shanghai, China; 12 Guangdong Provincial Key Laboratory of Genome Read and Write, Shenzhen, Guangdong China; 13 Shenzhen Engineering Laboratory for Birth Defects Screening, BGI Research, Shenzhen, Guangdong China

**Keywords:** Transcriptomics, Bioinformatics, Developmental biology, Cell signalling

## Abstract

The placenta, a temporary but essential organ for gestational support, undergoes intricate morphological and functional transformations throughout gestation. However, the spatiotemporal patterns of gene expression underlying placentation remain poorly understood. Utilizing Stereo-seq, we constructed a Mouse Placentation Spatiotemporal Transcriptomic Atlas (MPSTA) spanning from embryonic day (E) 7.5 to E14.5, which includes the transcriptomes of large trophoblast cells that were not captured in previous single-cell atlases. We defined four distinct strata of the ectoplacental cone, an early heterogeneous trophectoderm structure, and elucidated the spatial trajectory of trophoblast differentiation during early postimplantation stages before E9.5. Focusing on the labyrinth region, the interface of nutrient exchange in the mouse placenta, our spatiotemporal ligand–receptor interaction analysis unveiled pivotal modulators essential for trophoblast development and placental angiogenesis. We also found that paternally expressed genes are exclusively enriched in the placenta rather than in the decidual regions, including a cluster of genes enriched in endothelial cells that may function in placental angiogenesis. At the invasion front, we identified interface-specific transcription factor regulons, such as *Atf3*, *Jun*, *Junb*, *Stat6*, *Mxd1*, *Maff*, *Fos*, and *Irf7*, involved in gestational maintenance. Additionally, we revealed that maternal high-fat diet exposure preferentially affects this interface, exacerbating inflammatory responses and disrupting angiogenic homeostasis. Collectively, our findings furnish a comprehensive, spatially resolved atlas that offers valuable insights and benchmarks for future explorations into placental morphogenesis and pathology.

## Introduction

The placenta acts as the major organ of nutrient exchange between the mother and the embryo. It also serves as a vital fetal endocrine organ during pregnancy^1^. Placentation is a complex and dynamic process that is crucial for regulating communication and coadaptation between the mother and fetus^2^. Significant placental disruptions are always associated with various pregnancy complications and adverse fetal outcomes^3^. However, a profound understanding of key processes related to placentation, such as trophoblast development, fetal circulation establishment, and synchronized dialog between fetal-derived cells and the maternal endometrium^4^, remains a challenge. The gene expression pattern of the placenta has not been systematically characterized from a spatiotemporal perspective.

Previous single-cell and single-nucleus atlases have significantly enhanced our understanding of the cellular composition and cell-specific functions of the placenta, in both humans^5–7^ and rodents^8,9^. However, single-cell RNA sequencing (scRNA-seq) cannot capture multinucleated syncytiotrophoblasts (SynTs) and trophoblast giant cells (TGCs) in their mature state due to their large size^9,10^, nor could it provide spatial localization of diverse types of cells and gene expression patterns. Single-nucleus RNA sequencing (snRNA-seq), while capable of capturing multinucleated cells, cannot provide the complete transcriptome of the entire cell. Recently, several studies have used spatial transcriptome techniques to elucidate the locations of newly identified cell types^11^ and changes in gene expression in different functional regions^12^ of the human placenta. Nevertheless, most human placenta samples can be obtained from miscarriage samples in early pregnancy at only 6–14 gestational weeks or from labor and cesarean sections during late pregnancy^11,13^. Obtaining whole-layer human placenta samples across various developmental stages also presents technical and ethical challenges. Thus, there is a lack of resources for screening and observing the spatiotemporal characteristics of genes or pathways that are involved in placental development.

The mouse placenta is a model system that is widely used in exploring placenta biology due to its similarity to the human placenta in terms of haemochorial placentation and trophoblast-directed spiral artery remodeling^2,14^. A recent study used spatial transcriptomics to spatially define the dynamic functional decidual hub in mice focusing on endometrial decidualization^15^, but the spatial landscapes of the mouse placenta have not been elucidated. In rodents, the extraembryonic ectoderm (ExE) gives rise to the chorion and the ectoplacental cone (EPC) at ~E7.5^16^. The EPC was formerly divided into the ‘inner EPC’ and ‘outer EPC’, consisting of proliferative cells and relatively mature cells respectively^17,18^. However, the exact substructures of the EPC remain unclear. As the trophoblast lineage develops, the mouse placenta was gradually separated into two main regions — the junctional zone (JZ) and the labyrinth, serving distinct functions^19^. At ~E8.5, the fusion of the chorion and allantois during haemochorial placentation allows for the invagination of mesoderm­derived fetal vessels into the trophoblast layer, establishing the mouse placental labyrinth, which performs exchange functions similar to those of human chorionic villi^2,20^. Several signaling pathways, including the VEGF, Notch, Wnt, and Hedgehog signaling pathways, have been shown to play roles in placental angiogenesis and labyrinth development^21^. However, fully understanding the cellular interactions that involve these signaling pathways remains a challenge, as scRNA-seq failed to capture large trophoblasts, and the distance between ligand–receptor pairs was not fully considered in previous studies^8,9^. The JZ overlies the labyrinth and comprises glycogen trophoblasts (GlyT), spongiotrophoblasts (SpT), and various types of trophoblast giant cells, which are derived from EPC and mainly function in humoral secretion and vasculature remodeling^19^. Between JZ and decidua, the trophoblast lineage also gives rise to the interface of invasion, in which multiple types of immune cells, stromal cells, and trophoblasts collectively regulate decidual vascular development, vascular remodeling, and immune tolerance through complex interactions^15^. Nevertheless, there is a lack of research on the spatial characteristics of the decidua region closely adjacent to invasive trophoblasts across different stages.

Recently, we used the spatial transcriptome technology Stereo-seq to establish a high-resolution transcriptome map of mouse embryos, proving that gene expression landscapes and developmental lineages of organogenesis could be visualized with a large view at the single-cell level^22^. Here, Stereo-seq was used to establish the spatial transcriptomic landscapes of the mouse placenta during E7.5 to E14.5, spanning from trophoblast cell differentiation during early placental development to the formation of the placenta^23^, which covers the important stages of mouse placentation, such as chorioallantoic fusion at ~E8.5 and fetoplacental circulation establishment at ~E14.5^24^. We revealed the spatiotemporal characteristics of previously poorly understood subregions, such as the primitive EPC structure, labyrinth, and the interface of placental invasion, under physiological conditions. To investigate the mechanisms underlying abnormal placentation caused by adverse in-utero exposures, we explored the effects of a high-fat diet (HFD), which is a prevalent factor that is associated with pregnancy complications^25^, on spatial gene expression patterns during placentation. Furthermore, the spatial analysis revealed an unexpected compartmentalized expression pattern of imprinted genes in placentation, with paternally expressed genes mostly enriched in the placenta and maternally expressed genes mostly enriched in both the labyrinth and decidua. The atlas provides a valuable spatially resolved resource for studying placental development and advances the understanding of gene expression patterns within the uterus during placentation.

## Results

### Spatiotemporal transcriptomic atlas of mouse placentation with cellular resolution

**Fig. 1:**
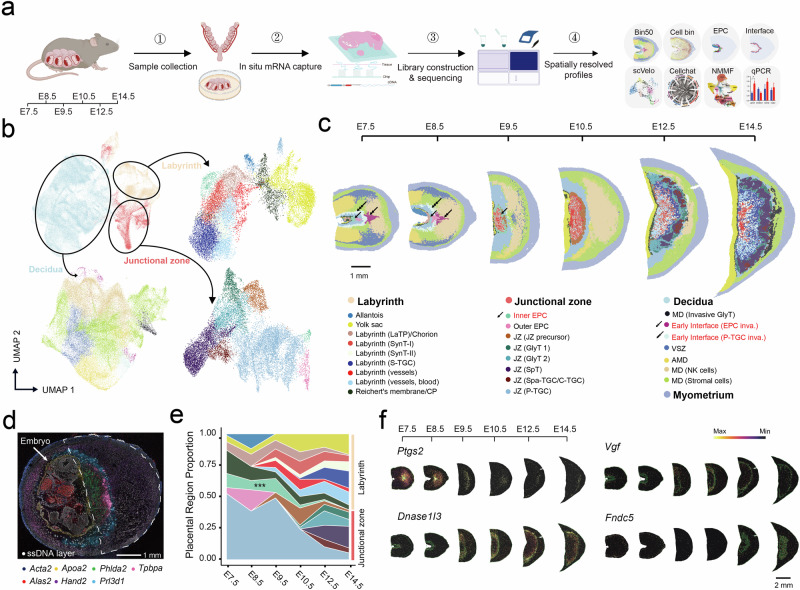
A spatiotemporal transcriptomic atlas of mouse placentation. **a** Schematic illustration of the workflow for this study. Sections of mouse uterine segments were collected at time points ranging from E7.5 to E14.5 for Stereo-seq. **b** UMAP plot showing bin 50 of the labyrinth, JZ, and decidua from eight placental sections at six stages. Bins are colored according to placental structure or subregion annotations that were established by unsupervised Louvain clustering and manual annotation steps. **c** E7.5 S1, E8.5 S1, E9.5 S1, E10.5 S1, E12.5 S1, and E14.5 S1 uterine sections are shown with 24 annotated subregions together with the myometrium. Arrows with one head, arrows with two heads and arrows with three heads indicate the inner EPC, the early interface (EPC inva.) and the early interface (P-TGC inva.), respectively. Bin colors indicating subregion annotations are the same as in **b**. LaTP labyrinth trophoblast progenitor, SynT syncytiotrophoblasts, CP chorionic plate, EPC ectoplacental cone, JZ junctional zone, GlyT glycogen trophoblast, SpT spongiotrophoblast, SPA-TGC spiral artery-associated TGC, C-TGC canal TGC, P-TGC parietal TGC, MD mesometrial decidua, VSZ vascular sinuses zone, AMD anti-mesometrial decidua. **d** Multicolor display showing the E10.5 S1 section; seven well-known markers and a ssDNA layer are indicated. **e** Quantification of the proportion of each subregion in the labyrinth and JZ captured at each developmental stage. All 13 uterine sections were used in this analysis. Colormaps are shown as in **c**. **f** Spatial visualization of the expression of the indicated genes in the placenta from E7.5 to E14.5.

The spatiotemporal transcriptome of mouse placentation was profiled using 13 whole uterine sections (including embryos) from C57BL/6J mice at different developmental stages (E7.5, E8.5, E9.5, E10.5, E12.5, and E14.5) (Fig. 1a and Supplementary Fig. S1a, b). The data can be accessed via our interactive data portal at https://db.cngb.org/stomics/mpsta/. For each section, we utilized the lasso function of StereoMap to segment tissues that contained placenta, decidua, and myometrium at the placental implantation site for further analysis (Supplementary Fig. S1b; Materials and methods). As a result, a total of 266,622 spots of bin50 were obtained from 13 sections, and the average gene number per spot ranges from 1135 to 3018 (Supplementary Fig. S1a, c, d). The transcriptome profiles that were obtained from sections in the same developmental period were highly consistent (Supplementary Fig. S1e), and they were also strongly correlated with published scRNA-seq data from the same developmental stage^8^, indicating the high reliability of the datasets (Supplementary Fig. S1f). Unsupervised spatially constrained clustering (SCC) of each section clearly distinguished the external boundaries of clusters that corresponded to major anatomic regions, including the labyrinth, JZ, decidua, and myometrium (Fig. 1b). To further explore functional regions across different stages, we integrated and co-embedded the bins corresponding to the labyrinth, JZ, and decidua from eight representative sections (Fig. 1b and Supplementary Fig. S2a, b; Materials and methods). Using canonical markers, a total of 24 subregions were ultimately annotated, including 7 from the labyrinth, 8 from the JZ, and 9 from the decidua (Fig. 1b, Supplementary Fig. S2c, and Tables S1, S2). We then remapped these annotated subregions to each section and established a spatiotemporal map of placentation containing the entire uterus (Fig. 1c). To verify our unbiased clustering and annotation results, typical markers of multiple subregions were examined, including *Acta2* in myometrium, *Alas2* in the labyrinth (vessels; blood), *Apoa2* in the yolk sac, *Hand2* in the decidua^26^, *Phlda2* in the labyrinth^27^, *Prl3d1* in polyploid parietal trophoblast giant cells (P-TGCs), and *Tpbpa* in the JZ^8,9^, which showed good consistency with their reported spatial localization (Fig. 1d). The remaining sections were annotated with the subregions identified above (Supplementary Fig. S2d, e; Materials and methods). In summary, we generated a comprehensive and highly reliable spatiotemporal transcriptomic atlas of mouse placentation.

Next, we investigated the dynamic changes and characteristics of the subregions in the placenta (Fig. 1e) and decidua (Supplementary Fig. S2f). In general, the subregions in the labyrinth and JZ underwent substantial and rapid changes, with a primary transition occurring from the chorion and EPC to labyrinth and JZ regions at ~E9.5 (Fig. 1e). Interestingly, the inner EPC, which was previously reported to localize to the interior side of the EPC at E7.5–8.5, migrated to the exterior side of the labyrinth and JZ at E9.5. These results uncovered the potential spatial trajectory of cells within inner EPC (Fig. 1c). On the decidual side, we revealed two distinct decidual subregions that were located proximal to the early maternal–fetal interface, and these regions were adjacent to the P-TGC and the EPC regions (Fig. 1c). These regions were named the “early interface” (P-TGC invasion) and “early interface” (EPC invasion), with the latter exhibiting elevated expression of *Ptgs2* and *Inhba* from E7.5 to E8.5 (Fig. 1c, f and Supplementary Fig. S2c). Moreover, we were able to effectively capture the transcriptomes of all multinucleated SynTs and P-TGCs at consecutive stages, which has rarely been achieved by previous scRNA-seq studies^8,9^ (Fig. 1c). Notably, we observed that *Dnase1l3*, which encodes secreted DNASE1-like nuclease that is capable of digesting DNA in maternal and fetal circulation^28^, was highly expressed in P-TGCs from E7.5 to E14.5 (Fig. 1f and Supplementary Fig. S2c). This indicates the potential role of P-TGCs in secreting DNase to degrade DNA from apoptotic fetal and placental cells during pregnancy in rodents. We also identified the expression of certain transcription factors (TFs) such as *Zfp42* in P-TGC with high regulon activities, apart from the previously known *Fosl1* and *Hand1* (Supplementary Fig. S2g, h and Table S3)^18,29^. By investigating the spatiotemporal dynamics of gene expression across different subregions, we identified a subset of genes with high regional or temporal specificity in expression, providing valuable spatiotemporal insights into the molecular events underlying placentation (Supplementary Fig. S3a and Table S4). For example, we identified the expression of *Ptprn* and *Fdx1*, related to steroid hormone synthesis^30,31^, in the E10.5 P-TGCs, which are known for hormone synthesis functions^32^ (Supplementary Table S5). Additionally, genes that function in sprouting angiogenesis, including *Ncoa3* and *Fuz*, were enriched in SynT-II at E14.5 (Supplementary Table S5). We also identified the placental localization of certain hormone-encoding genes, such as the expression of *Vgf* in P-TGCs (encoding VGF, expressed before E7.5 and until E14.5) and *Fndc5* in sinusoidal TGCs (S-TGCs) (encoding Irisin, expressed after E12.5) (Fig. 1f).

Finally, we further performed image-based cell segmentation and spatial mapping of the placenta and decidua in all sections using public scRNA-seq datasets^8,33^, which profiled transcriptomes of mouse placenta from E7.5 to E14.5 and mouse uterus at E6.0, to analyse their expression landscape at the single-cell level (Supplementary Fig. S4a; Materials and methods). Several recently identified progenitor or precursor cell types^8^ were successfully mapped to their expected locations (Supplementary Fig. S4a), and the dynamic changes in these cell populations were also shown accordingly (Supplementary Fig. S4b, c). Previous single-cell analysis revealed the trajectory of labyrinth trophoblast development across E9.5–E14.5, indicating that labyrinth trophoblast progenitor (LaTP) cells transition into SynT-II cells, while LaTP2 cells give rise to SynT-I cells^8,9^. In our study, by calculating adjacent cell proportions for LaTP and LaTP2 cells at E10.5, we observed the adjacent relationship between LaTP2 and SynT-I as well as a higher proportion of trophoblast stem cells (TSCs), ExE cells, and LaTP cells near SynT-II, which supports previous findings (Supplementary Fig. S4d, f)^9^. In summary, we successfully established the MPSTA with Stereo-seq, which enables us to explore gene function from a spatial perspective.

### Spatial identification of EPC layers and their trajectory across E7.5–E9.5

**Fig. 2:**
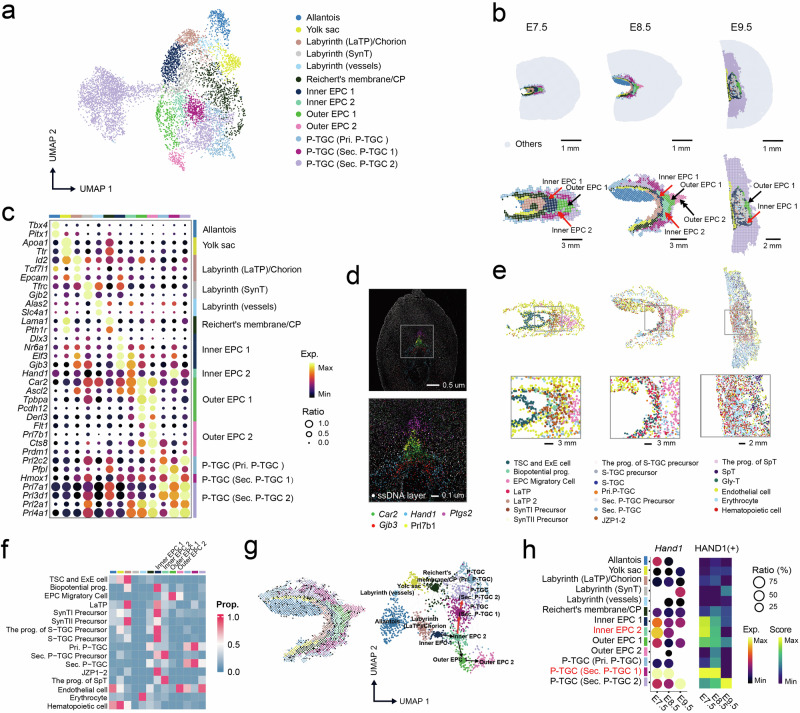
Transcriptomic landscape during early trophoblast development. **a** UMAP plot showing the integrative clustering and annotation of bin50 from the labyrinth and JZ; E7.5 S1, E7.5 S2, E8.5 S1, E8.5 S2, and E9.5 S1 sections were used for this analysis. **b** E7.5 S1, E8.5 S1, and E9.5 S1 uterine sections are shown with (top) and without (bottom) trophoblast structures. **c** Bubble plot showing the expression profiles of representative marker genes. Gene labels are displayed in the left panel, and corresponding clusters are annotated and colored in the top and right panels. Colors from black to yellow indicate low to high gene expression levels. **d** Multicolor display of representative markers and magnified images of the ectoplacental cone regions in section E8.5 S1. **e** Top: spatial visualization of deconvoluted cell types in the sections in **b**. Bottom: magnified images showing the cellular composition in the regions in the square of the upper panel. **f** Heatmap showing scaled proportions of the cell types in each cluster in section E8.5. S1. **g** Left: spatial RNA velocity streamlines visualization of the directional flows. Right: RNA velocity PAGA graph predicting the developmental trajectory of the ectoplacental cone and P-TGCs in section E8.5 S1. Bins are colored by cluster identity, as in **a**. **h** Transcript expression of *Hand1* in each cluster across early stages and its median regulon activity score (RAS) among all the clusters.

The EPC, which serves as the initial indication of placentation in rodents and the origin of various types of trophoblasts^17^, is a heterogeneous trophectoderm structure that exists from E7.5 to E8.5. In the previous section, the distribution of EPC subregions was observed within the mature placenta at E9.5 (Fig. 1). To gain a detailed understanding of the spatial heterogeneity and developmental connections of the EPC subregions, we pooled the bins of five representative uterine segments from E7.5 to E9.5 and removed the decidua and myometrium regions to perform unsupervised clustering (Fig. 2a and Supplementary Fig. S5a; Materials and methods). We obtained a total of 13 distinct subregions to establish an elaborate spatial partitioning, particularly within the EPC and P-TGC regions (Fig. 2a). Interestingly, after remapping the annotated subregions to spatial coordinates, the EPC was divided into distinct layers (Fig. 2b), which were named inner EPC1, inner EPC2, outer EPC1, and outer EPC2, in order from the inner to outer positions. Three distinct layers were also identified within the P-TGC region: the primary P-TGC, secondary P-TGC1, and secondary P-TGC2. Notably, the secondary P-TGC1 region was located between the inner EPC and the secondary P-TGC2 region.

The expression patterns of key markers within the EPC region revealed interesting spatial insights (Fig. 2c, Supplementary Fig. S5b, and Table S6). The proliferative marker *Gjb3* exhibited high expression levels in both the inner EPC1 and inner EPC2 (Fig. 2c, d). The genes expressed in inner EPC1 and inner EPC2 were enriched in tissue morphogenesis and regulation of cell‒cell adhesion, respectively (Supplementary Fig. S5b), suggesting their roles in cell proliferation and development. In contrast, the outer EPC 1 and outer EPC2 regions exhibited high expression of the JZ markers *Car2* and *Tpbpa*^34^, indicating their identity as JZ precursors (Fig. 2c, d). We also observed that the outer EPC1 predominated over the JZ at E9.5, except for P-TGC (Fig. 2b) and exhibited high expression of *Ascl2*, a JZ-enriched TF at early stages^35^; these results emphasize the close developmental connections of these regions. Intriguingly, *Sox4*, a TF expressed in human extravillous trophoblasts (EVTs) and associated with EVT differentiation^36,37^, exhibited high expression and regulon activity in the outer EPC2 region at E8.5 and the maternal–fetal interface at E9.5 (Supplementary Fig. S5d). Additionally, the outer EPC2 region specifically expressed invasive trophoblast markers, such as *Prl7b1*, *Prdm1*, and *Cts8*^38^ (Fig. 2c, d and Supplementary Fig. S5b, c), and genes that are involved in the regulation of angiogenesis and extracellular matrix organization (Supplementary Fig. S5c, e). Therefore, we propose that the outer EPC2 region may be enriched with early invasive trophoblasts and might closely interact with the decidua at E8.5. Further analysis of cellular compositions within the EPC region showed a significant proportion of EPC migratory cells in the outer EPC regions. In contrast, the inner EPC region, particularly the inner EPC1 region, was predominantly composed of progenitor and precursor cells (Fig. 2e, f). We also observed that S-TGC precursors and bipotential progenitor cells were primarily located within the inner EPC1 region, and these cells have the potential to differentiate into labyrinth trophoblasts in the mature placenta. Conversely, the precursors of secondary P-TGCs were primarily enriched in the inner EPC2 region, consistent with the higher expression of the TGC transcription factor *Hand1* in this region (Supplementary Fig. S5c). For the P-TGC region, a few markers were identified, including *Prl2c2* in primary P-TGCs before E9.5 as well as *Hmox1* and *Hand1* in secondary P-TGC1 before E9.5 (Fig. 2c and Supplementary Fig. S5b, c). Taken together, these results revealed the specific regional composition and markers of the EPC and P-TGC.

Furthermore, spatial RNA velocity analysis was conducted to determine the trajectory across different regions and extract topological information (Fig. 2g; Materials and methods). The spatial RNA velocity analysis revealed strong sequential directional flows from the inner EPC1, inner EPC2, and outer EPC1 to the outer EPC2, indicating migration and differentiation directions within these regions (Fig. 2g). In addition, we identified another path that flows from the inner EPC2 region towards the secondary P-TGC1 region and later transitions to the secondary P-TGC2 region (Fig. 2g). These results suggested that secondary P-TGC1 might be the precursors of mature secondary P-TGCs in the relative outer layer. The migration path was consistent with our observations of a shift in the expression and regulon activity of Hand1, a key TF required for TGC differentiation^18^, in these regions during different stages of development (Fig. 2h). Thus, our results supported a model in which EPC cells migrate from the bottom of the cone and gradually differentiate into mature cells. Simultaneously, precursors of P-TGCs migrate from the inner EPC towards both sides and further develop into P-TGCs.

### Spatiotemporal ligand–receptor dynamics uncovered key regulators of labyrinth development

**Fig. 3:**
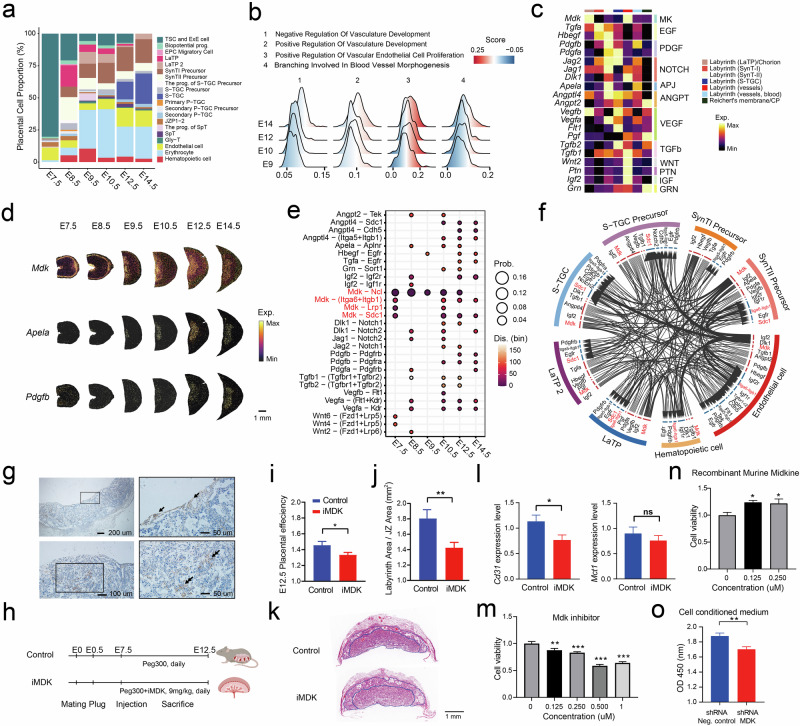
Systematic regulation events during labyrinth development. **a** Temporal dynamics of labyrinth cell proportion (allantois, yolk sac, and Reichert’s membrane/Chorionic plate (CP) were excluded). **b** Ucell score variation across different stages in sets of genes that are involved in vascular-related biological processes. **c** Spatial expression profiles of key hormones from several signaling pathways. **d** Visualization of the spatial expression of *Mdk, Apela*, and *Pdgfb*. **e** Significant spatially restricted ligand–receptor interacting pairs were identified in the labyrinth at different stages. **f** Ligand–receptor gene pairs involving key growth factors that mediate cell–cell communications in the labyrinth as identified in scRNA-seq datasets. The outside ring shows cell types, and the inside ring shows the details of each interacting ligand–receptor pair. Both are color-coded. The width of the line and arrowheads inside are scaled to indicate the relative expression levels of the ligand and receptor, respectively. **g** IHC staining showing the location of MDK expression in the chorionic plate and SynT of the labyrinth (indicated by an arrow). **h** Schematic diagram showing the process of iMDK treatment. **i** Placental efficiency as expressed by the ratio of fetal weight to placental weight (*n* = 24 for control, *n* = 41 for iMDK). **j** Measurements of the labyrinth area and JZ area and the ratio of the labyrinth area to the JZ area (*n* = 8 control, *n* = 14 iMDK). **k** Representative image of a cross-section through the labyrinth and JZ in a mid-section of a mouse placenta. The blue solid line outlines the labyrinth and JZ that was measured in (**k**). **l** Real-time quantitative polymerase chain reaction (RT-qPCR) analysis of the expression of markers of SynT-I (*Mct1*) and endothelial cells (*Cd31*). RT-qPCR data were normalized to the reference gene *Hprt*. **m**–**o** The CCK-8 assay was used to evaluate the proliferation of endothelial cells treated with iMDK (**m**), recombinant murine MDK (**n**), and cell-conditioned medium collected from *shRNA-MDK*-treated trophoblast cells and shRNA-Neg-treated trophoblast cells (**o**). The data were presented as the means ± SEM. *P < 0.05, **P < 0.01, ***P < 0.001, ns not significant.

During haemochorial placentation, the development of trophoblast cells and the expansion of the fetal vasculature are essential for facilitating the exchange function of the labyrinth^20^. We observed a substantial increase in the numbers of erythrocytes and hematopoietic cells in the chorion and labyrinth, which was accompanied by a decrease in the numbers of TSCs and ExE cells, especially between E8.5 and E9.5. This represents the critical window for the maturation of trophoblasts and the establishment of fetal circulation (Fig. 3a). In the “Labyrinth (vessels)” region, the pathways related to positive regulation of angiogenesis were increasingly enriched from E9.5 to E14.5, which was consistent with the sustained expansion of placental vasculature during the mid to late stages of gestation^21^ (Fig. 3b). Interestingly, the activation of negative regulation pathways was observed at ~E14.5, indicating the potential shift from high activation of angiogenesis to a certain degree of equilibrium after the labyrinth becomes fully functional.

To further elucidate the key factors involved in labyrinth development, we examined the spatial expression of growth factors and their interactions (Fig. 3c–f). We observed placenta-derived *Apela*, an endogenous ligand of the apelinergic system^39^ in the Labyrinth (SynT-II), heparin-binding growth factor *Midkine* (*Mdk*) in the Labyrinth (LaTP)/Chorion (Fig. 3c, d and Supplementary Fig. S6a), and the enrichment of *Pdgfb*, an endothelial cell-derived factor, in the peripheral labyrinth near the JZ beginning at E10.5 (Fig. 3d and Supplementary Fig. S6a). Furthermore, an analysis of spatially constrained cell‒cell communication revealed a complex and dynamic local interaction network between these factors and their nearby receptors (Fig. 3e and Supplementary Fig. S6b; Materials and methods), which was supported by the public scRNA-seq data^8^ (Fig. 3f). For instance, significant communication between SynT-II-derived *Apela* and endothelial cell-derived *Aplnr* was also observed locally between SynT-II and the vessel region (Fig. 3e, f and Supplementary Fig. S6b). Intriguingly, our analysis revealed strong local interactions associated with *Mdk* in the labyrinth across different developmental stages (Fig. 3e and Supplementary Fig. S6b). *Mdk*, a heparin-binding growth factor, was reported to be involved in cell growth, migration, and angiogenesis in tumors^40^. The expression of *Mdk* was predominantly observed in SynT-II cells (Supplementary Fig. S6c) and significantly interacted with the *Itga6*–*Itgb1* in endothelial cells (Fig. 3f and Supplementary Fig. S6c, d). Immunostaining and PCR assays also confirmed the localization of MDK in the chorionic plate and trophoblasts (Fig. 3g and Supplementary Fig. S6e, f), suggesting that MDK might be involved in labyrinth development. To investigate the roles of MDK expressed by syncytiotrophoblasts in labyrinth development, we intraperitoneally injected pregnant mice with an Mdk inhibitor (iMDK) once a day from E7.5 to E12.5. The effectiveness of the inhibitor was confirmed using western blotting (WB) and immunochemistry (IHC) assays (Fig. 3h and Supplementary Fig. S6g, h). The inhibition of MDK resulted in a decrease in fetal weight and placental efficiency at E12.5 (Fig. 3i and Supplementary Fig. S6i). Additionally, there was a decreased ratio between the labyrinth area and the JZ area (Fig. 3j, k and Supplementary Fig. S6j), which is consistent with the placental phenotype reported under hypoxia conditions during gestation^41^, indicative of abnormal placental blood flow and restricted placental development. Considering the region of SynT-II in which *Mdk* was expressed and potential interactions of *Mdk* with adjacent endothelial cells and SynT-I, we performed PCR assays, finding that iMDK treatment significantly decreased the expression of *Cd31*, a marker gene for endothelial cells, without affecting the markers of SynT-I and SynT-II in placenta (Fig. 3l and Supplementary Fig. S6k). Further, we found that iMDK significantly decreased the proliferation of mouse primary umbilical vein endothelial cells (MUVEC) (Fig. 3m) while recombinant murine MDK increased their proliferation (Fig. 3n). Additionally, iMDK inhibited endothelial cell migration (Supplementary Fig. S6l–m), whereas recombinant murine MDK promoted their migration (Supplementary Fig. S6n, o). Treating endothelial cells with cell-conditioned medium from trophoblast cells with knocked-down *Mdk* expression (Supplementary Fig. S6p), we observed that endothelial cell proliferation was inhibited (Fig. 3o). In summary, our findings revealed spatially restricted ligand–receptor interactions in the labyrinth, suggesting that *Mdk* may play a role in regulating labyrinth development, potentially through its effects on endothelial cells.

### Compartmentalized expression pattern of imprinted genes during placentation

**Fig. 4:**
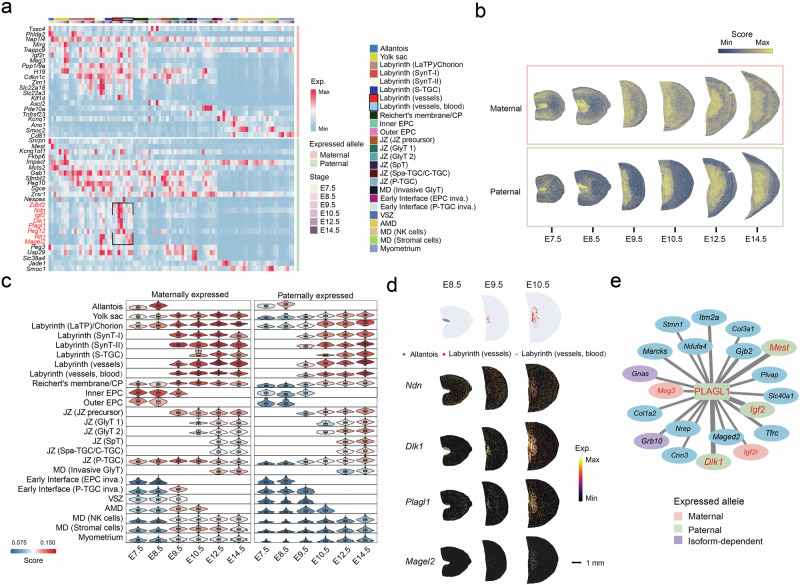
Spatial distribution of imprinted genes in the placenta. **a** Spatiotemporal expression of reported imprinted genes in the placenta^45^. **b**, **c** Ucell scores of all paternally and maternally expressed imprinted genes captured by spatial transcriptomics within the imprinted gene set are visualized in spatial subregions (**b**) and aggregated for different subregions across stages (**c**). **d** The top panel shows the locations of the allantois, labyrinth (fetal vessels), and labyrinth blood space at early stages, followed by the spatial expression profiles of the imprinted genes *Ndn*, *Dlk1*, *Plagl1*, and *Magel2*. **e** Gene regulatory networks of the top 20 target genes of *Plagl1* at E14.5; colors indicate different imprinted statuses. Genes highlighted in red have had their allelic expression reported^45^. Font size and width of lines connecting each target gene are proportional to their network importance scores in the regulon.

Imprinting genes have been previously proven to play crucial roles in placentation^2^, and several paternally expressed genes are involved in placental angiogenesis, including *Mest*^42^, *Plagl1*^43^, *Igf2*^44^. In this case, by taking advantage of our spatiotemporal transcriptomic data, we first utilized spatial transcriptome data to analyze the expression patterns of previously reported mouse imprinted genes (www.geneimprint.com) in the placenta, and we highlighted genes showing a parental bias in expression reported by Gigante et al.^45^ (Fig. 4a and Supplementary Fig. S7a). Interestingly, gene set enrichment analysis revealed a compartmentalized expression pattern of these allele-specific imprinted genes as captured by spatial transcriptomics. Paternally expressed genes were predominantly enriched in the placenta, whereas maternally expressed imprinted genes were more abundant in both the labyrinth and decidua, but underrepresented in the JZ (Fig. 4a–c). A similar imprinting pattern has also been observed at the maternal–fetal interface of the human placenta^11^ (Supplementary Fig. S7b). Paternally expressed orthologous genes are most active in VCT, fF1, and fF2, while maternally expressed orthologous genes are also prominent in EVT and glycogen cells (Supplementary Fig. S7c). In particular, we observed a cluster of paternally expressed imprinted genes that were specifically enriched in the fetal vessel region of the labyrinth, including *Zdbf2, Ndn*, *Igf2*, *Dlk1*, *Plagl1*, *Peg12*, *Rtl1*, and *Magel2* (Fig. 4a and Supplementary Fig. S7a). Previous studies have demonstrated the crucial involvement of *Igf2*, *Rtl1*, and *Plagl1* in the expansion of the placental microvasculature^43,44,46^. However, the localization and specific roles of other genes, such as *Ndn* and *Magel2*, have not been clearly illustrated. Based on our data, the expression patterns of these genes were comparable to that of *Dlk1*, which was enriched in the allantois before E8.5 and later in the labyrinth (vessel) subregion (Fig. 4d). Furthermore, by utilizing published scRNA-seq data, we also observed notable enrichment and increased expression of *Ndn* and *Magel2* in endothelial cells throughout various developmental stages^8^ (Supplementary Fig. S7d, e). IHC assays confirmed the expression of *Ndn* in the vessels of both the chorionic plate and labyrinth (Supplementary Fig. S7f). Additionally, we found that the top 20 target genes of the TF *Plagl1* included a range of imprinted genes located in endothelial cells, including paternally expressed genes, such as *Dlk1*, *Mest*, and *Igf2*, and maternally expressed genes, such as *Meg3* and *Igf2r*, highlighting the significant role of *Plagl1* in placental angiogenesis^43^ (Fig. 4e, Supplementary Fig. S7d, and Table S7). Taken together, our results defined a compartmentalized expression pattern of imprinted genes during placentation and suggested that a group of paternally expressed genes may be involved in the intricate network of placental angiogenesis.

### Identification of interface-specific TF regulons that are involved in the maintenance of pregnancy

**Fig. 5:**
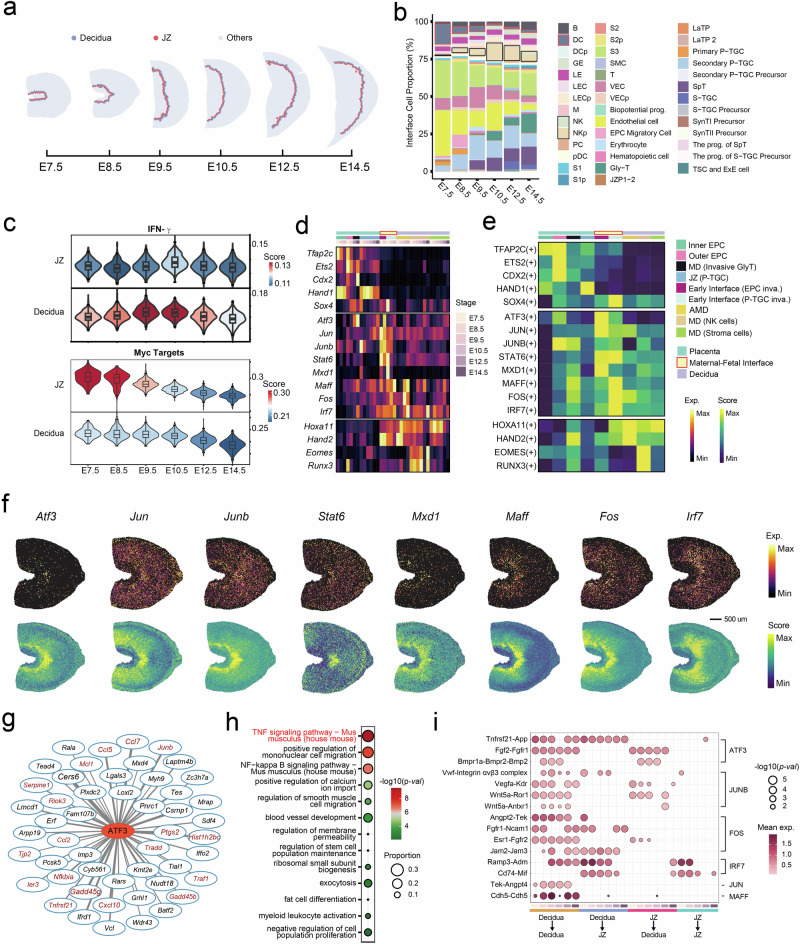
Transcriptomic landscape at the maternal–fetal interface. **a** Schematic of the isolated maternal–fetal interface layers across all stages. The decidua and JZ regions of the interface are shown in different colors. **b** Cell type composition of the isolated maternal–fetal interface across all stages. **c** Distribution of gene set scores is shown for Hallmark Interferon Gamma Response and Hallmark Myc Targets across all developmental stages. **d**, **e** Spatiotemporal expression profiles of selected transcription factors in each cluster (**d**) and their median RAS (**e**). **f** Spatial visualization of the expression (top) and RAS (bottom) of multiple transcription factors at E8.5. **g** Gene regulatory networks of *Atf3* at E8.5 S1 as visualized by Cytoscape. The top 50 target genes with the highest network importance scores are shown. Font size and width of lines connecting each target gene are proportional to their network importance scores in the regulon. Genes that are involved in the TNF signaling pathway are labeled in red. **h** Enriched pathways of the 50 top target genes of *Atf3* at E8.5. S1. **i** Interactions of selected ligand–receptor pairs between the decidua and JZ region of the isolated maternal–fetal interface across all six developmental stages.

During the invasion, trophoblasts and decidual cells undergo functional changes and adaptations orchestrated by various TFs to ensure the maintenance of normal pregnancy and immune homeostasis in the uterus^47,48^. While some in vitro experiments have demonstrated potential transcriptional regulatory activities that might occur at the maternal–fetal interface, such as the ability of trophoblast cells to induce the expression of *Stat6* in T cells, the highly activated TFs at the interface have not been systematically observed. To explore the cell compositions and in situ transcriptome of the early maternal–fetal interface, we designated the interface boundary, which consisted of three bin50 layers on both sides near the boundary (Fig. 5a; Materials and methods). On the decidua side of the boundary, the numbers of natural killer (NK) cells and proliferating natural killer (NKp) cells peaked at ~E10.5 (Fig. 5b) with coordinated activation of the interferon (IFN)-γ pathway, which mediates the spiral arterial modification of uterine natural killer cells^49^ (Fig. 5c). From E7.5 to E8.5, we observed a high proportion of dendritic cells (DCs), which highlighted their role in the initialization of the decidual response to embryo implantation^50^ (Fig. 5b and Supplementary Fig. S8a). As for interface trophoblasts, we observed a gradual decrease in the enrichment of the *Myc* pathway, which is related to placental development (Fig. 5c), and was consistent with the differentiation and maturation of trophoblasts. We also evaluated the cell composition and Gene Ontology enrichment in the early interface (EPC invasion) and early interface (P-TGC invasion) regions identified in Fig. 1 (Supplementary Fig. S8a–c and Tables S8, S9). The “early interface (EPC invasion)” region was involved in cytolysis and positive regulation of inflammatory responses, while the “early interface (P-TGC invasion)” region was associated with the regulation of peptidase activity as well as positive regulation of angiogenesis. Considering that this area is adjacent to P-TGC, which serves as invasive trophoblasts, the regulation of protease activity at specific local sites may be associated with the regulation of trophoblast invasion and vascular remodeling in the decidua. Taken together, our data presented the cellular composition and gene enrichment in the interface of placental invasion, in both the functional regions and localized regions with a defined distance at the interface.

To gain further insights into the regulation of the transcriptome at the interface, we examined interface-specific TFs that displayed high expression and regulon activity in the subregions near the interface, including *Atf3*, *Jun*, *Junb*, *Stat6*, *Mxd1*, *Maff*, *Fos*, and *Irf7* (Fig. 5d). Public scRNA-seq data^8^ were used to further confirm their specific expression in immune cells and decidual stromal cells (Supplementary Fig. S8d). Spatial visualization of the expression and regulon activities of some TF genes suggested their sustained and active roles at the interface as gestation progressed, such as *Maff* and *Fos* in decidual stromal cells (Fig. 5f and Supplementary Fig. S8e). In contrast, the expression of *Atf3* was primarily enriched in the early interface from E7.5 to E8.5, and then, its expression shifted from the decidua to the labyrinth in subsequent stages (Supplementary Fig. S8f).

Most of the interface-specific TFs identified in our study have been reported as dysregulated in pregnancy complications, especially in recurrent spontaneous abortion (RSA) and preeclampsia, which suggests their potential roles in regulating the inflammatory response or placental invasion. For example, increased mRNA levels of *Fos*^51^, *Jun*, *Junb*^52^, and *Atf3*^53^ were observed in placental or decidual tissues of women with miscarriage. Among these TFs, we observed the enrichment of *Atf3* in immune cells (Supplementary Fig. S8d), particularly in macrophages and DCs (Supplementary Fig. S8g) in the early decidual interface, and the target genes of *Atf3* were significantly enriched in the TNF and NF-kappa B signaling pathways (Fig. 5g, h and Supplementary Tables S10, S11). Given the positive correlation between TNF-α levels and miscarriage risk in both animals^54^ and humans^55^, we further explored the association between *Atf3* and the TNF signaling pathway. *Atf3* was colocalized with *Ptgs2*, a target gene of *Atf3* that is involved in TNF signaling, at the early interface (Supplementary Fig. S8g, h). Previous in vitro studies using chromatin-immunoprecipitation analysis have reported that ATF3 negatively regulates *Tnfα* and *Ptgs2* and subsequently modulates inflammatory responses in macrophages^56,57^. Considering the known role of ATF3 in regulating inflammatory responses in macrophages^58^ and its localized regulon activity at the interface, we hypothesized that ATF3 may have a function at the early maternal–fetal interface. Furthermore, we explored the possible local interactions regulated by these interface-specific TFs (Fig. 5i; Materials and methods). We observed an interaction between *Tnfrsf21*, a predicted target gene of *Atf3* involved in TNF signaling, and *App* at the interface. This interaction has been reported to be involved in cell necroptosis at the interface^59^. Collectively, we hypothesized that ATF3 might play a role in maintaining a balanced inflammatory response at the early maternal–fetal interface by regulating the TNF signaling pathway, though it still needs more investigation. Additionally, ATF3 may also regulate the expression of other TFs, including *Junb* in the immune cells^58^. Among the target genes of *Junb*, we also observed many inflammation-related genes that are involved in the interactions identified at the interface, such as Wnt5a–Ror1 and the Vwf–Integrin aVb3 complex. Taken together, we identified several interface-specific TF regulons that might be involved in the maintenance of pregnancy, and our data suggested the potential role of ATF3 in maintaining a balanced inflammatory response at the interface.

### Maternal HFD specifically affects the interface by elevating inflammatory responses and disrupting angiogenesis

**Fig. 6:**
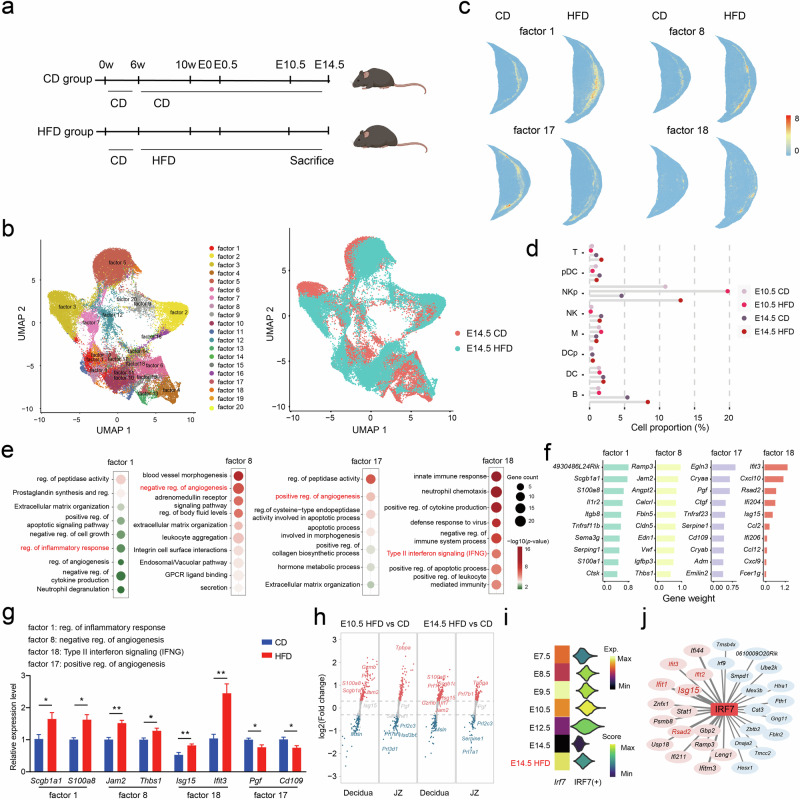
Effect of high-fat diet consumption on regulation at the maternal–fetal interface. **a** Schematic diagram showing the HFD treatment schedule and time points for sampling. **b** UMAP representation of 20 factors in the CD and HFD groups at E14.5. **c** Spatial distributions of factors 1, 8, 17, and 18 are shown, and colors from light blue to red indicate low to high coefficients for each bin50. **d** Proportions of immune cell types at the isolated maternal–fetal interface; E10.5 S1, E10.5 S2, E10.5 S3, H10.5 S1, E14.5 S1, E14.5 S2, and H14.5 S1 sections were used for this analysis. **e** Functional enrichment based on the top 50 weighted genes associated with factors 1, 8, 17, and 18. **f** Top ten genes defining the indicated NNMFs (factors). **g** RT-qPCR analysis of the expression of genes associated with factor 1 (*Scgb1a1* and *S100a8*), factor 8 (*Jam2* and *Thbs1*), factor 18 (*Isg15* and *Ifit3*) and factor 17 (*Pgf* and *Cd109*). **h** Genes that were differentially expressed in the decidual and JZ of the maternal–fetal interface are shown. **i** Gene expression level and regulon activity of *Irf7* in the decidua of the interface region. **j** Top 15 target genes of *Irf7* in the CD and HFD groups at E14.5 are shown, and genes that were associated with factor 18 are labeled in red.

HFD intake, a prevalent adverse intrauterine exposure for women of reproductive age, is associated with the development of various pregnancy complications, including preeclampsia, gestational diabetes mellitus, and preterm delivery, as well as fetal growth restriction^60,61^. Pregnant mice were fed either a control diet (CD) or an HFD for 4 weeks before and during pregnancy. Uterine segments were then collected for sequencing by Stereo-seq at E10.5 and E14.5 (Fig. 6a and Supplementary Fig. S9a, b). The upregulated genes in the HFD group in the yolk sac region were enriched in functions related to lipoprotein transport (Supplementary Fig. S9c, d), likely reflecting the sense and response of the placenta and fetus facing the nutritional status of the mother. The overall regional composition and cell proportions in the HFD group did not show significant changes compared to the CD group (Supplementary Fig. S9e). However, Principal Component Analysis (PCA) on the cell Moran’s I score, a spatial autocorrelation statistic, clearly distinguished the HFD group from the CD group (Supplementary Fig. S9f). Notably, decidual immune cells, endothelial cells, and stromal cells located near the interface, including NKp, VEC, S1p, and S2p cells exhibited the most pronounced changes in self-localization profiles and displayed variations in the neighborhood cell proportions of most cells, which indicates the potential impact of HFD on the interface (Supplementary Fig. S9g, h).

We then compared the data from the CD and HFD groups at E14.5 by nonnegative matrix factorization (NNMF), and 20 factors representing distinct patterns of gene expression changes were identified (Fig. 6b, Supplementary Fig. S10a, and Table S12). Notably, many factors (factors 1, 8, 11, 17, 18, and 19) were enriched at the maternal–fetal interface, particularly in the mesometrial decidua regions, which showed enrichment of invasive trophoblast cells and NK cells (Supplementary Fig. S10b). Among these factors, factors 1, 8, and 18 were predominantly expressed in the placentas of the HFD group, while factor 17 was predominantly distributed in the placentas of the CD group, corresponding to the upregulation and downregulation of genes after HFD exposure, respectively (Fig. 6c and Supplementary Fig. S10c). An examination of cellular components at the defined interface boundary (as shown in Fig. 5) revealed that the placentas of the HFD group had higher proportions of proliferating NK cells than the placentas of the CD group at both E10.5 and E14.5 (Fig. 6d and Supplementary Fig. S9e). Concordantly, factor 1 and factor 18 were associated with pathways that are involved in the regulation of the inflammatory response (Fig. 6e, f and Supplementary Table S13). Specifically, factor 18 was characterized by the expression of several genes encoding interferon-induced proteins (such as *Ifit1*, *Ifit2*, and *Ifit3*) and pathways involved in type II interferon signaling (*Cxcl10*, *Ccl2*, and *Isg15*) (Fig. 6e, f), which have been reported to play important roles in vascular remodeling and maintenance of pregnancy at the interface^62–66^. Then, RT-qPCR was performed to validate *Ifit3* and *Isg15* upregulation (Fig. 6g). Additionally, top genes involved in factor 8 and factor 17 were both enriched in pathways associated with angiogenesis (Supplementary Table S13). Factor 8 was associated with negatively regulating angiogenesis, while factor 17 was enriched in pathways related to the positive regulation of angiogenesis (Fig. 6e). Consistently, PCR assays revealed decreased expression of proangiogenic genes, including *Pgf* and *Cd109*, and increased expression of genes that are associated with the negative regulation of angiogenesis, such as *Jam2* and *Thbs1*, in the placentas of the HFD group (Fig. 6g). An analysis of genes that were differentially expressed at the interface also revealed similar changes in the expression of some genes, such as *Jam2* (Fig. 6h). Overall, our analysis revealed that the impact of HFD on the interface transcriptome primarily involves the induction of inflammatory responses and the disruption of angiogenesis regulation.

Furthermore, we observed that the top genes in these factors, which were differentially expressed at the interface, were largely target genes of those interface-specific TFs, especially for *Fos*, *Maff*, and *Irf7*. For instance, the expression and regulon activity of *Irf7*, which is a TF that was shown to be highly expressed at the interface (Fig. 5), was significantly enhanced at the interfaces of the HFD group at E14.5 (Fig. 6i), and its top target genes included several top genes belonging to factor1 (*S100a8*), factor8 (*Jam2*), and factor18 (*Isg15*) (Fig. 6j and Supplementary Table S14). These results suggest that activation of IRF7 and aberrant interferon signaling might mediate the effect of HFD on the expression of genes that are associated with inflammatory responses at the interface and thus contribute to adverse perinatal outcomes^67^. We also observed significant changes in the interactome within the decidua caused by maternal HFD exposure, as evidenced by PCA clustering of the interactome between decidua and JZ regions (Supplementary Fig. S10d). Further investigation revealed that interactions involving *Angptl4*, *Vegfd*, and *Thbs1* exhibited higher probabilities within the decidual regions or between the decidual and JZ regions in the HFD sections (Supplementary Fig. S10e). This suggests that these interactions may contribute to the regulation of angiogenesis homeostasis in the HFD group.

### The distribution of heart defect-associated genes in the placenta

Except for HFD-related placenta developmental disorders, placental abnormalities have been identified as a potential factor contributing to developmental defects such as congenital heart disease (CHD)^68^. To gain further insights into the potential impacts of the placenta on fetal heart development, we examined the expression of a dozen genes, identified by whole exome sequencing in families affected by CHD^69^ or characterized by Deciphering the Mechanisms of Developmental Disorders (DMDD, https://dmdd.org.uk) consortium^70,71^, in the placenta. Sampling the entire uterus allowed us to simultaneously observe the expression patterns of genes in both the fetal heart (*Myh6*) and placenta (*Krt8*) (Supplementary Fig. S11a). As expected, we observed the expression of several typical gene markers in mouse heart tissue, including *Myh6*, *Myh7*, and *Actc1* (Supplementary Fig. S11b, c and Tables S16, S17). Interestingly, several genes that are associated with heart defects, including *Atp11a*, *Camsap3*, and *Flvcr2*, exhibited higher expression levels in the placenta than in the fetal hearts from E10.5 to E14.5 (Supplementary Fig. S11b–d). Recent research has proposed that *Atp11a*-induced SynT-I defects could be a potential link between placental function and developmental heart disorders^72^. In our study, we identified several genes, including *Arid1b, Flvcr2*, *Camsap3*, and *Rpgrip1l*, that were expressed in locations similar to *Atp11a* in the SynT-I and SynT-II regions. Furthermore, we also observed enrichment of *Ssr2*, *Chst11*, *H13*, *Sh3pxd2a*, and *Braf* in the GlyT layer (Supplementary Fig. S11b–d). Our results systematically revealed the expression patterns of genes potentially involved in fetal heart development within the placenta. However, the functions of these genes during placental development and their roles in fetal heart development need further study.

## Discussion

Despite the important roles of the placenta in the maintenance of pregnancy, the spatiotemporal gene expression pattern during placentation has been poorly characterized. Previous applications of spatially resolved transcriptomic technologies have mainly focused on separated placental tissues in early pregnancy^11^. In this study, we established the most comprehensive spatiotemporal transcriptomic maps of mouse placentation from E7.5 to E14.5. Our atlas provides an intrauterine-wide perspective of spatial gene expression and delivers more comprehensive transcriptomic profiles of cell types compared to previous single-cell atlases. We elucidated the characteristics of critical functional regions, including the EPC, labyrinth, and interface of placental invasion. Particularly, at the site of placental invasion, spatial transcriptomic profiling identified interface-specific TF regulons that are involved in the maintenance of pregnancy. We also revealed that maternal HFD exposure in utero specifically affects the interface by enhancing inflammatory responses and disrupting the regulation of angiogenesis. Furthermore, spatial analysis revealed unexpected compartmentalized expression patterns of imprinted genes during placentation. Overall, our atlas provides a valuable spatially resolved resource for studying placental development and advances our understanding of gene expression patterns during placentation.

A major strength of our study is the inclusion of multinucleated and large cells, such as mature SynTs and P-TGCs, and the establishment of a more reliable data resource, which has not been achieved by previous single-cell sequencing studies. The mouse placenta contains a large number of multinucleated cells and giant trophoblast cells; however, only a limited number of SynT precursors and P-TGCs prior to E8.5 can be captured by previous scRNA-seq studies due to the large sizes of these cells. Furthermore, subgroups of P-TGCs were not precisely defined in previous scRNA-seq studies since *Prl4a1*, the typical marker of secondary P-TGCs, was expressed in “primary P-TGCs”^8,16^. With our spatial transcriptome data, a substantial number of P-TGC-enriched regions were identified and further stratified into three subregions. In addition to these classifications, we also observed the expression of *Dnase1l3*, *Vgf*, and genes that are involved in steroid biosynthetic processes in P-TGCs, which indicates the paracrine and endocrine functions. Interestingly, *Vgf* has been reported to be especially expressed in neuroendocrine cells after E10.5^73,74^. Considering more and more literatures indicating the crosstalk between the placenta and fetal brain through pathways such as secretion^72,75,76^, placental neuroendocrine factors like VGF might be involved in this connection^77^. These findings enhance our understanding of the functions of specific trophoblasts.

Another strength of our study lies in the characterization of the substructures of the EPC, the maternal–fetal interface boundary, and the labyrinth. As the initial indication of placentation in rodents, the EPC was previously divided into the “inner EPC” and “outer EPC” layers, each composed of distinct cell types, including progenitors of different cell lineages^17,18,78^. However, the spatial organization of the EPC is still unclear. Here, we showed that the EPC can be divided into four subregions with distinct cell states and gene markers. We noticed that the progenitors of secondary P-TGCs were distributed in the inner EPC2. Secondary TGCs were thought to arise from *Tpbpa*^+^ EPC precursors^79^, but recent studies also suggested that secondary P-TGCs may have other precursor cells^8,18^. Here, we observed the presence of *Tpbpa*^–^ secondary P-TGC precursors in the inner EPC2 region and showed their spatial differentiation trajectory. In addition, we found that the outer EPC2 expressed high levels of invasive trophoblast markers and regulons. Our analysis suggests that *Tpbpa*^+^ invasive trophoblasts (outer EPC2) and other JZ cells (outer EPC1) were spatially separated at ~E8.5. The spatial proximity to the decidua suggests that these trophoblasts of the outer EPC2 might participate in the early initiation of communication between the placenta and the decidua. Recently, the inner decidua region has been identified as a vascular hub that contains immune decidual stromal cells^15^. Similarly, we also found that the genes specifically enriched in the outer EPC2 region were involved in the regulation of angiogenesis. Thus, these results imply a potential shared regulation of decidual vascular development by both trophoblasts in the outer EPC2 and decidual stromal cells at the early interface.

The labyrinth is the exchange interface in the placenta of rodents where trophoblasts and endothelial cells are enriched. Using published single-cell data, we determined the locations where *Pdgfb* and *Mdk* are expressed, as well as their roles in angiogenesis and proliferation during labyrinth development. *Pdgfb* has been reported to prevent the premature differentiation of hematopoietic stem and progenitor cells^80^. The interactions between *Pdgfb* in endothelial cells and *Pdgfrb* in haematopoietic cells were observed at ~E12.5, which is consistent with the period of the haematopoietic niche in the placenta (Fig. 3e, f). In addition, the expression of *Pdgfb* in the labyrinth near the boundary area might explain why immature haematopoietic cells in the haematopoietic niche near the JZ are more abundant than erythroid clusters near the chorionic plate at E10.5 and E11.5^81^. MDK is reported to play a role in hypoxia-induced angiogenesis^82^. Human umbilical vein endothelial cells release significant amounts of soluble MDK under hypoxia, and exogenous MDK can induce neovascularization in vitro^82^. Fan et al. reported the expression of *Mdk* in the chorion at E9.5, but its cellular location and function remain unclear^83^. In our dataset, *Mdk* was continuously expressed in the chorion and labyrinth from E7.5 to E14.5. Spatially restricted cell communication analysis predicted the interactions between *Mdk* with *Itga6*–*Itgb*, *Sdc1*, and *Ncl*. A decreased ratio between the labyrinth area and the JZ area was observed in mice treated with iMDK, resembling what is observed under conditions of abnormal placental blood flow or hypoxia^41^.

At the interface of trophoblast invasion, we identified TFs expressed in immune cells or decidual stromal cells at the early interface, employing a different strategy compared to previous single-cell analyses^84,85^. The abnormal expression of these TFs in pregnancy complications^51–53^ suggests the potential roles of these TFs in the maintenance of pregnancy. Among the interface-specific TFs, we focused on *Atf3* because of its potential roles in regulating inflammatory responses via TNF signaling. In previous studies, ATF3 was shown to be involved in the transition from villous cytotrophoblasts (VCTs) to cytotrophoblast cell columns (CCCs) in trophoblasts and decidualization in stromal cells^11,86^. Here, our results suggest a potential role of *Atf3* in maintaining immune homeostasis, as its expression is enriched in immune cells at the early interface. Additionally, we found that the predicted target genes of *Atf3* and their interactions were associated with TNF signaling, which is linked to RSA^54^. For instance, *Tnfrsf21*, predicted to be a top target gene of *Atf3*, has also been reported to be upregulated in the endometrium of patients with recurrent pregnancy loss^59^. Furthermore, it has been reported that ATF3 negatively regulates the transcription of TNF-α in mice challenged with lipopolysaccharide^57^. Based on our data, we hypothesized that ATF3 might suppress TNF signaling and help maintain a balanced inflammatory response at the early maternal–fetal interface. However, the functions of ATF3 and other interface-specific TFs in the pathological processes of abnormal pregnancies require further investigation.

To investigate the mechanisms underlying abnormal placentation caused by adverse in-utero exposures, we compared the spatial transcriptomic characteristics of placentas in the HFD and normal groups. HFD is a major risk factor for pregnancy complications in women of reproductive age^60,61^, and obesogenic diets have been reported to cause deficiencies in-utero–placental vascularization^87^ and a reduction in uterine blood flow volume^88^. NNMF analysis revealed abnormal activation of inflammatory pathways, such as the interferon signaling pathway, and disruption of the regulation of angiogenesis in the decidual region of the HFD group, rather than other regions. Considering the overlap between the top target genes of *Irf7* and the most upregulated genes in factor 1 and factor 18, we speculate that interface-specific upregulation of *Irf7* (shown in Fig. 5) may play a crucial role in HFD-induced interferon signaling activation at the interface. IRF7 is involved in the IFN response^89,90^, and interferon signaling pathway-related genes, including *Irf7*, *Isg15*, and *Ifit1* (belonging to factor 1), have been reported to be activated in endometrial inflammation and diseases including recurrent pregnancy loss^59,91,92^. After HFD exposure, the expression of *Irf7* was upregulated in adipose tissues, liver and muscle and led to systemic inflammation^93,94^, and *Irf7* may exert similar effects in the decidual region. Additionally, genes such as *Jam2* and *Pgf*, which were included in factor 8 and factor 17, may contribute to the effect of HFD on the decidual vasculature.

Another strength of this spatial atlas is its unique utility for observing the expression patterns of specific gene sets in the whole placenta and throughout the entire uterus, such as the imprinted genes and candidate genes affecting fetal development, which might be closely associated with placental function. Notably, we observed an unexpected compartmentalized expression pattern of imprinted genes during placentation, with paternally expressed genes enriched in the placenta and maternally expressed genes enriched in both the labyrinth and decidua. Based on kinship theory, paternally and maternally expressed genes are thought to promote and suppress fetal growth, respectively^95^. However, the functions of some genes do not align well with these predictions, which might be due to the complex regulatory network among imprinted genes^96^. Our findings offer a new spatial view to observe these genes, such as the localization of *Ndn* in fetal vessels in the labyrinth, for the first time. The labyrinth is responsible for nutrient transport, and Sandovici et al. reported that the endothelial cells-derived paternally expressed IGF2 plays a critical role in the expansion of the placental microvasculature^44^. Consistently, increased IGF2 levels in the umbilical cord and elevated expression of *DLK1*, *MEST*, *PLAGL1*, and *NDN* in placental tissues have been associated with increased birth weight in large for gestational age babies^44,97^. Thus, the enrichment of paternally expressed genes, especially in endothelial cells, might suggest their crucial roles in the allocation of resources and regulation of angiogenesis. The enrichment of maternally expressed genes in the interface adjacent to the maternal uterus suggests that these genes might be involved in immune tolerance, vascular remodeling, and the regulation of fetal and placental growth. The enrichment of imprinted genes in different regions offers insights into their functions, such as the role of PLAGL1 during placental vascular development^43^.

CHD is recognized as the most common birth defect, but 55% of cases have unknown causes^98^. Previous studies have shown that the functions of genes expressed in the placenta can impact the development of the fetal heart and brain^72,76^. Notably, we observed that some CHD-associated genes, such as *Raf1*, and some genes that are associated with heart morphology, including *Sh3pxd2a* and *Arid1a*, were expressed in the placenta, especially in the SynT and GlyT. Among the genes highly expressed in the placenta, *ARID1A* has been reported to negatively regulate trophoblast cell proliferation and invasion and is upregulated during preeclampsia^99^. In addition, ARID1B, which has high structural similarity to ARID1A, has also been associated with aberrant methylation of the WNT2 signaling in the placenta during preeclampsia^100^. Nevertheless, it remains unclear whether fetal heart development is regulated by placental *Arid1a*. Additionally, in the landscapes that include the entire uterus and the fetus, *Sh3pxd2a* was shown to be expressed in GlyT cells but not in fetal cells. SH3PXD2A has been previously reported to be potentially involved in the pathogenesis of preeclampsia and trophoblast function^101^. However, *Sh3pxd2a* knockout models exhibited embryonic heart defects, specifically ventricle septal defects, without significant placental abnormalities^71^. Our study provides a foundational atlas for the identification of potential pathogenic genes in CHD. Further research is needed to confirm the link between heart development and genes expressed in the placenta and to explore the underlying mechanisms.

In conclusion, we offered a spatiotemporal landscape of transcriptional dynamics in the developing placenta, particularly in several critical functional regions, including the EPC, labyrinth, and the interface of placental invasion. The atlas provides a valuable spatially resolved resource for the investigation of gene expression patterns during placentation. However, this study has limitations. Although our dataset includes at least two placentas from different dams at each developmental time point, the relatively limited number of samples and sections weakens the robustness of the atlas. Future studies could include more time points and use multiple section-based 3D reconstruction to enhance the dataset. Furthermore, while we identified the locations of interface-specific TF regulons and placenta-expressed genes implicated in heart defects, greater focus should be placed on the functional exploration of these genes using transgenic mouse models.

## Materials and methods

### Software and algorithms

**Table:** 

Toolkit	available at	version
SAW	https://github.com/BGIResearch/SAW	V5.1.3
Python	https://www.python.org/	V3.9.13
Spateo	https://github.com/aristoteleo/spateo-release	V1.0.2
Scanpy	https://github.com/theislab/scanpy/	V1.9.1
Cell2location	https://github.com/BayraktarLab/cell2location	V0.1.3
pySCENIC	https://github.com/aertslab/pySCENIC/	V0.12.1
ScVelo	https://github.com/theislab/scvelo	V0.2.5
Dynamo	https://github.com/aristoteleo/dynamo-release	V1.1.0
Tangram	https://github.com/broadinstitute/Tangram	1.0.3
Stereopy	https://github.com/STOmics/Stereopy	V0.11.0
R	https://www.r-project.org/	V4.2.2
Seurat	https://satijalab.org/seurat/	V4.3.0
ComplexHeatmap	https://github.com/skvark/opencv-python	V2.15.3
Cellchat	https://github.com/sqjin/CellChat	V2
CellphoneDB	https://github.com/genostack/CellphoneDB	V4

### Ethics statement

Ethical approval for the mouse experiments was provided by the Ethics Committee of Shanghai Model Organisms (license number IACUC2021-0038) and the Institutional Review Board of BGI-Shenzhen (BGI-IRB 22037, BGI-IRB A23043).

### Uterus collection and sample preparation for Stereo-seq

C57Bl/6J mice were maintained under SPF conditions at the Shanghai Model Organisms Center. To obtain samples of uterus tissues at specific stages, female mice between 8–12 weeks of age were mated with male mice between 10–16 weeks of age in the afternoon. The next morning, female mice with vaginal plugs were transferred to new cages; this time point was designated as E0.5. Female mice were sacrificed at E7.5, E8.5, E9.5, E10.5, E12.5, and E14.5, and the entire uterus was harvested and then divided into individual implantation sites. Dissections were performed on ice and in cold PBS. At later gestational time points, including E12.5 and E14.5, a certain amount of amniotic fluid was removed. Uterine segments were washed with PBS to remove blood from the surface. Tissues were then placed in cryomoulds and immediately embedded in optimal cutting temperature compound (Sakura, 4583). The frozen samples were stored at –80 °C for further use. For Stereo-seq, cryosections were cut sagittally at a thickness of 10 μm using a Leica CM1950 cryostat. Developmental stages for each segment were later confirmed again by morphological observations. The section that included the maximum sagittal section was used for Stereo-seq. An extra sagittal section next to each Stereo-seq section was also used for hematoxylin and eosin (H&E) staining.

### Stereo-seq library preparation and sequencing

The processes of library construction and spatial transcriptomics capture was carried out following a previously described protocol^22^. The tissue sections on the Stereo-seq chips were incubated at 37 °C for 6–12 min and subsequently fixed in methanol. In situ reverse transcription, amplification, library construction, and sequencing steps were then performed as previously described^22^.

### In vivo functional validation of the role of MDK in labyrinth development

iMDK was purchased from MCE (HY-110171). Pregnant female mice were intraperitoneally injected with 200 µL of a solution containing iMDK (9 mg/kg) and PEG300 once a day from E7.5 to E12.5. The control group received PEG300 alone. Pregnant mice were sacrificed at E12.5, and the fetuses and their placentas were collected and weighed after the yolk sacs and endometrium were removed. Dissections were performed on ice and in cold PBS using fine-pointed forceps. The placental tissues were stored at −80 °C for further experiments. Placentas that were obtained for H&E staining or IHC assays were fixed with 4% paraformaldehyde for at least 24 h and embedded in paraffin blocks.

### Cells and cell culture

Mouse primary trophoblasts and MUVEC were isolated from placenta of pregnant C57Bl/6J mice and purchased from Shanghai Zhong Qiao Xin Zhou Biotechnology Co., Ltd. Cells were seeded in DMEM (Gibco) containing 10% fetal bovine serum (FBS; TransGen Biotech) and antibiotics (100 units/mL penicillin and 100 μg/mL streptomycin) incubated in a humidified atmosphere of 5% CO_2_ at 37 °C.

### CCK-8 assay of endothelial cell proliferation

Cell viability was analyzed by Cell Counting Kit-8 (C0048, Beyotime Biotechnology) in accordance with the CCK-8 assay protocols. Cells were seeded at a density of 3 × 10^3^ per well in 100 µL of medium in 96-well plates (Corning, USA). After 48 h, 10 µL of CCK-8 was added to the cells and incubated at 37 °C for 3 h. A microplate reader (Tecan Spark, Tecan Group, Ltd.) was used to measure the absorbance of each well at 450 nm.

### Wound-healing assay

MUVEC were seeded in 6-well plates at a density of 2 × 10^5^ per well. Wounds were generated by scratching the cell layer with 1-mL sterile plastic pipette tips and rinsed in the culture medium. The final images were acquired with a microscopy (IX73, Olympus, Japan).

### Lentivirus infection

The lentiviral shRNA expression vectors for mouse *MDK* (shRNA*-MDK*) and a nontargeting shRNA expression vector (shRNA-neg) serving as a negative control were purchased from Obio Technology. The lentiviral shRNA expression vectors were transfected into cells with 6 µg/mL polybrene.

### Preparation of trophoblast-derived conditioned medium

To collect the conditioned medium, primary trophoblast cells were seeded at a density of 2 × 10^5^ per well in 2 mL of medium in six-well plates (Corning). After 24 h of transfection using shRNA*-MDK* and shRNA-neg, the medium was collected and stored at –80 °C for further experiments.

### HFD model establishment

To establish the maternal HFD models, six-week-old female C57BL/6J mice were randomly assigned to receive a control diet (CD, 10% calories from fat, Xietong Pharmaceutical Bioengineering, Jiangsu, China) or HFD (60% calories from fat, D12492; FBSH Bio-Pharmaceutical, Shanghai, China), and both groups were given ad libitum access to food and drinking water. After 4 weeks of consuming these diets, females were mated with C57BL/6J males. Pregnancy was determined by the presence of a copulation plug (E0.5), and dams were maintained on their assigned diet throughout gestation. Uterine segments were harvested on E10.5 and E14.5 for Stereo-seq, and placental tissues were isolated and stored at –80 °C for further experiments.

### IHC

Sections (3-µm thick) were deparaffinized twice in xylene for 15 min each and then incubated in a series of ethanol solutions (2× pure ethanol, followed by 1× 85% ethanol and 1× 75% ethanol for 5 min each) in PBS. Antigen retrieval was performed by boiling in 10 mM Na-citrate pH 6.0 buffer, and 3% H_2_O_2_ was used to quench endogenous peroxidases. After cooling, the slides were washed three times with PBS for 5 min each, followed by blocking in PBS supplemented with 0.5% BSA and 0.1% Tween-20 for 30 min at room temperature. Next, the samples were incubated with diluted primary antibodies overnight at 4 °C. After three washes in PBS for 5 min each, the tissue sections were incubated in diluted secondary antibodies at room temperature for 50 min. Primary antibodies were detected with horseradish peroxidase-conjugated secondary antibodies, and visualization was achieved by incubating the slides with diamino benzidine (DAB) (Dako, K5007). The nuclei were counterstained with hematoxylin. The antibodies used included anti-Mdk (1:400; Abcam, ab52637), anti-Necdin (1:200; Abcam, ab227908), and HRP-labeled goat anti-rabbit (Dako, K5007) antibodies. Finally, the sections were dehydrated and sealed by neutral balsam. The staining was observed with a light microscope (XSP-C204, CIC, China) and a slide scanner (KFBIO, Ningbo, China).

### cDNA synthesis and qPCR

RNA was extracted using the FastPure Cell/Tissue Total RNA Isolation Kit V2 (Vazyme, Nanjing, China). cDNA was synthesized from RNA using the RT Reagent Kit and gDNA Eraser (RR047A, Takara, Shiga, Japan). qPCR was performed on the QuantStudio 5 Flex system (Applied Biosystems, Foster City, CA, USA) using SYBR Premix EX Taq (RR420A, Takara Bio, Shiga, Japan). The samples used in the research were run in triplicate. The threshold cycle values were used to calculate relative gene expression levels, and values were normalized to *Hprt* transcripts. Forward and reverse primer sets are listed in Supplementary Table S15.

### Western blotting assay

Proteins were extracted from placental tissues using RIPA lysis buffer (P0013B; Beyotime, Shanghai, China), and protein concentrations were determined with a BCA protein assay (Thermo Fisher). Then, the proteins were mixed with 5× loading buffer (Beyotime, P0015L) and boiled for 5–7 min. The samples were separated using SDS-PAGE at 80–120 V for 90 min and transferred onto PVDF membranes at 220 mA for 90 min. The membranes were blocked in TBS-T (150 mM NaCl, 10 mM Tris-HCl, pH 8.0, and 0.5% vol/vol Tween-20) with 5% skim milk powder for ~1 h at room temperature. Then, primary antibodies were added and incubated overnight at 4 °C, and secondary antibodies were added and incubated for 1 h at room temperature. The protein signals were detected via Western Chemiluminescent HRP Substrate (Millipore, WBKLS0500). The signals were visualized using an enhanced chemiluminescence system (Amersham Imager 600, GE Healthcare Life Sciences, Pittsburgh, PA, United States). The antibody used was an anti-MDK antibody (1:1000; Abcam, ab52637).

### Stereo-seq raw data processing

The Stereo-seq Analysis Workflow (*SAW*) software suite [https://github.com/BGIResearch/SAW] was used to process the raw data as previously described^22^. In brief, the coordinate identity (CID) sequences (read 1, 1–25 bp) were matched with the records of CID-coordinate key-value pairs to determine the spatial location on the tissue section (allow 1 mismatch). Molecular identity (MID) sequences (read 1, 26–35 bp) containing N bases or with quality scores below 10 for more than 2 bases were filtered out. cDNA sequences (read 2, 100 bp) were aligned to the reference genome (GRCm38) by *STAR*^102^. The spatial total MID count expression matrices, together with the exon and intron expression matrices, were generated from the above pipelines.

### Unsupervised SCC

Unsupervised SCC was conducted on each section using *Spateo*^103^ with the bin50 count matrices, and then, the clusters were annotated based on anatomical partitions and marker genes. Count matrices were first log-normalized, and a transcriptomic neighbor graph was calculated by S*canpy*^104^. For each Stereo-seq section, a 30-nearest expression neighbor graph and an eight-nearest spatial neighbor graph were combined as input for *Louvain* clustering by *Spateo* with tuned resolutions. Furthermore, cluster-specific markers were identified by the *rank_genes_groups* function of *Scanpy*.

### Integrated analysis of multiple sections

To construct placenta-wide developmental maps with time points ranging from E7.5 to E14.5, we integrated and co-embedded bin50 from representative sections at each time point using *Scanorama*^105^ together with Scanpy. The bin50 UMI count matrices for the decidua, JZ, and labyrinth of each section were first log-normalized, genes with highly variable expression were identified, and PCA was performed for dimension reduction with *Scanpy*. *Scanorama* was then used to calculate integrated embeddings from different sections, which were subsequently used to compute merged neighbor graphs. Louvain clustering was then conducted, and the results were visualized in *umap* projection; and resolutions of 2.4, 2.0, and 1.8 were selected for the decidua, JZ, and labyrinth, respectively. All of these bin 50 were ultimately annotated in 25 clusters based on previously well-defined marker genes. Similarly, for the early trophoblast development maps, we integrated and co-embedded bin50 of both the JZ and labyrinth regions from representative sections at time points ranging from E7.5 to E9.5; a resolution of 2.4 was chosen, and 13 clusters were defined.

### Spatial RNA velocity analysis

We utilized scVelo^106^ for RNA velocity analysis, following the tutorial that is available at https://scvelo.readthedocs.io/en/stable/. From the five sections (E7.5 S1, E7.5 S2, E8.5 S1, E8.5 S2, and E9.5 S1), we extracted unspliced and spliced RNA for each bin50 using SAW. The extraction of the Labyrinth and JZ bins was based on subregion classification. The resulting data matrix was then processed by scVelo, which involved normalizing the expression, selecting feature genes, and performing PCA dimension reduction. Default parameters were used to estimate the kinetic parameters and genewise RNA velocity vectors, which were subsequently projected to the physical space. We visualized the streamlines of only the EPC and P-TGC trajectories. To enhance the understanding of gene expression dynamics, we extended the velocity-inferred directionality to construct a PAGA graph embedded in UMAP.

### Mapping subregions for sections

After we identified subregions for the eight representative sections, we applied *Tangram*^107^ mapping for the remaining sections from the control and HFD groups. Briefly, the subregions were mapped to each section using the representative section at the same stage as the reference. Cell mapping mode was used for the *map_cells_to_space* function of Tangram.

### Cell segmentation

The C*ellCut* functionality of the *SAW* workflow was used to identify the cell coverage area on the nuclear ssDNA image. After obtaining the mask results of cell segmentation, a cell bin GEF was generated, including expression information of the cells, such as the coordinates of the centroid, boundary coordinates, expression of genes, and cell area. The *gef* files were further converted to loom format using the *generate_loom* function in *Stereopy* [https://github.com/STOmics/Stereopy].

### Gene regulatory network analysis

Gene regulatory networks were inferred from Stereo-seq bin matrices according to the *pySCENIC* protocol^108^. The databases used for *pySCENIC* were downloaded from https://resources.aertslab.org/cistarget/databases/. Bin50 matrices were used to perform *SCENIC* analysis. Coexpression modules were inferred, pruned, and quantified from the count matrix by *GRNBoost2*, cisTarget, and *AUCell*, respectively. Together with the physical coordinates, *RASs* were visualized across developmental stages by *ComplexHeatmap*^109^.

### Regulon downstream analysis

For each stage/section, regulon specificity scores (*RSSs*) were calculated across clusters. The *RAS* of each regulon was first normalized to the probability distribution. Organs were then represented as a vector of binary labels (target organ: 1; others: 0) and normalized to a probability distribution. These steps were followed by Jensen‒Shannon divergence (*JSD*) calculations to measure the differences between the two normalized probability distributions with a range from 0 to 1. Last, JSD scores were converted to *RSSs* using the formula: *RSS* = *1 – sqrt (JSD)*. A higher *RSS* score indicates increased enrichment of the regulon in the corresponding cluster. We ranked regulons in each cluster based on their *RSSs* and selected the top 5 or top 10 as cluster-specific regulons.

### NNMF analysis of the HFD placenta sections

The NNMF method was employed to compare CD and HFD sections. First, the gene expression matrices from E14.5 CD and HFD were integrated, followed by normalization and scaling. The *nmf* function of the *NMF* package was then used to process the scaled data, with parameters set to *rank* = *20, method* = “*snmf/r*”*, and seed* = “*ndsvd*”. This yielded two matrices: genes*factors and factors*cells. Factors with significant differences in cell contributions between CD and HFD, and genes with high contributions in these factors were further analysed and visualized.

### Differential tests for HFD sections

HFD sections were first mapped with the subregion annotation as mentioned above. All the sections at E10.5 and E14.5 were first SCTransform-normalized using the *Seurat* package by a scale factor of 5000 and then merged separately. *FindMarkers* with *recorrect_umi* = *False* was then applied to identify significantly up- or downregulated genes in each subregion (*abs(avg_log2FC)* > 0.25 and *p_val_adj* < 0.05).

### Interface segmentation

An in-house script was used to extract the JZ and decidua interface region at the maternal–fetal interface. Briefly, bin50 with one side (up, down, left, right) facing decidua regions and the other side facing trophoblast regions were extracted and defined as decidua layer 1 or JZ layer 1. Then, the area was expanded to decidua or trophoblasts by steps of 50 and 100, namely, layer 2 and layer 3. A total of six layers were defined as the interface area that included the decidua interface and JZ interface, and these layers were further used to determine bin–bin communication or cell proportions.

### Cell–cell communication analysis in the labyrinth and the maternal–fetal interface region

*CellChat*^110^ and *CellPhoneDB*^5^ were applied separately to investigate bin–bin communications in the labyrinth and the maternal–fetal interface region. For the labyrinth communication analysis, we constrained the communication to occur only within the bin100 distance in *Cellchat*; thus, we only considered communication between closely located bins within the labyrinth. For the maternal–fetal interface region, we first extracted the bins from the JZ and decidua interface and converted mouse genes to human genes. *CellPhoneDB* was then used to identify ligand–receptor interactions that were enriched between the JZ interface and the decidua interface based on the coordinated expression of a receptor and ligand. Biologically relevant interactions were manually prioritized after filtering nonsignificant pairs.

### Deconvolution of scRNA-seq data by *Cell2location*

For cell type mapping analysis of trophoblasts and decidua cells, we separately downloaded scRNA-seq data from the recently published GSE156125 dataset^8^ and obtained data from He et al.^33^. After obtaining the gene expression matrix after cell segmentation, we split the spatially resolved cell bins into trophoblast and decidua groups according to their localization. The trophoblast cell bins were aligned to the reference cell types of the corresponding stage in GSE156125 using the principled Bayesian model in cell2location^111^. Decidual cell bins were similarly mapped to the cell types from the study by He et al.^33^. After mapping, the trophoblast cell bins and decidua bins were then merged to obtain a complete map of placental sections with a cell type-level resolution.

### Calculation of the proportions of different cell types at the placenta and cluster/interface

Combining the deconvolution results from both the trophoblast and decidua cell bins, the proportions of different cell types across the whole placenta were calculated as the number of cells of each type in a cell bin (e.g., NKp cell) divided by the total number of cells in the placenta. To calculate the proportion of each cell type at the interface or in a specific cluster, the bin50 cluster/interface annotation was first transferred to the cell bin according to coordinate mapping using the *st.dd.set_domains* function in *Spateo*, and then the proportions of cell types in specific clusters or at the interface were calculated as the number of cells of each type in a cell bin of the cluster/interface (e.g., biopotential progenitor in inner EPC1) divided by the total number of cells in the clusters/interfaces (e.g., inner EPC1).

### Spatial cell distribution analysis

The *dyn.tl.neighbors* functionality of *Spateo* was utilized to calculate spatial nearest neighbors for each cell type, with 20 cell neighbors set up for each cell. The *st.tl.cellbin_morani* function was used to first calculate cell counts in each bin50, followed by the calculation of Moran’s I score for each cell type in each section, which was then used for PCA.

### Analysis of mouse imprinted genes and heart defect-associated genes in the placenta

Genes that are associated with defects in heart development according to studies by Perez-Garcia et al.^71^ and Szot et al.^69^ were investigated in our study. We also downloaded imprinted genes from https://www.geneimprint.com/site/genes-by-species.Mus+musculus for further analysis. The expression levels of each gene in a subregion at each stage were SCTransform-normalized and aggregated using *Seurat*, further scaled and visualized with the R package *ComplexHeatmap*.

## Supplementary information

Supplementary information

Supplementary Tables

## Data Availability

All raw data generated in this study have been deposited to CNGB Nucleotide Sequence Archive under the accession number CNP0004966 (https://db.cngb.org/search/project/CNP0004966/). Our Stereo-seq dataset can be visualized by our interactive data portal (https://db.cngb.org/stomics/mpsta/). Additional data, including raw and filtered feature-spot matrices and nucleic acid dye staining images of each section, can be accessed from https://db.cngb.org/stomics/project/STT0000055.
