# Supplementary material for: A spatiotemporal transcriptomic atlas of mouse placentation
Source: Cell Discov. 2024 Oct 22;10:110. doi: 10.1038/s41421-024-00740-6 (PMC11496649; doi:10.1038/s41421-024-00740-6)
Supplement: Supplementary file 1 — Supplementary information [file 41421_2024_740_MOESM1_ESM.pdf]

A spatiotemporal transcriptomic atlas of mouse placentation

Yanting Wu[1,2,3,4], Kaizhen Su[2,5,6], Ying Zhang[7,8], Langchao Liang[9,10], Fei Wang[7,], Siyue Chen[1], Ling Gao[1], Qiutong Zheng[1], Cheng Li[1], Yunfei Su[1], Yiting Mao[1], Simeng Zhu[11], Chaochao Chai[9,10], Qing Lan[7], Man Zhai[7], Xin Jin[7], Jinglan Zhang[1,3,4], Xun Xu[7,12], Yu Zhang[1], Ya Gao[7,8,13], Hefeng Huang[1,2,3,4,5,6]

These authors contributed equally: Yanting Wu, Kaizhen Su, Ying Zhang, Langchao Liang

Correspondence: Hefeng Huang (huanghefg@hotmail.com); Ya Gao (gaoya@genomics.cn); Yu Zhang (zhang_yu_sfy@fudan.edu.cn); Yanting Wu (yanting_wu@163.com).

The PDF file includes:

Supplementary Figs. S1 to S11

Captions Supplementary Tables S1 to S17

Supplemental Figures

Supplementary information, Fig. S1

a

Stat \ Stage	E7.5	E8.5	E9.5	E10.5	E10.5 HFD	E12.5	E14.5	E14.5 HFD
# Sections	2	2	2	3	1	2	2	1
# Total bin50	21986	24138	27970	54143	19560	40651	52862	25312
# Total genes	20321	19375	19391	20924	21943	21094	20894	22024
# Average genes	3018	1741	1391	1689	2614	1650	1135	1754
# Average UMIs	11743	5058	4076	5095	9292	4456	3046	5702

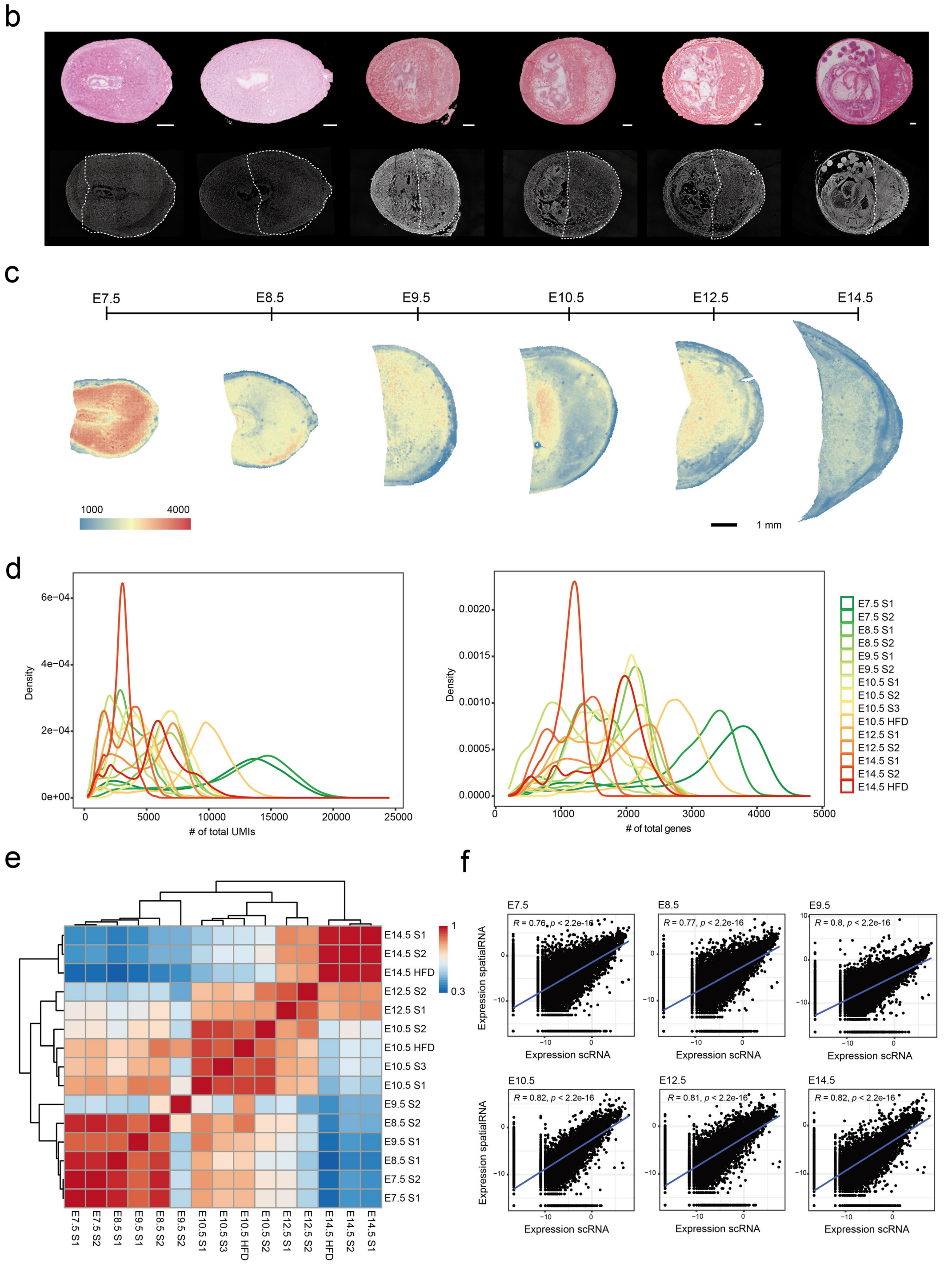

Supplementary information, Fig. S1: Quality control of Stereo-seq data

(a) Statistics for all chips used in this study. (b) ssDNA and H&E staining of the 6 uterine sections including E7.5 S1, E8.5 S1, E9.5 S1, E10.5 S1, E12.5 S1 and E14.5 S1. All scale bars indicate 1 mm. (c) Spatial visualization of gene numbers at bin50 resolution at each stage. (d) Distribution of detected UMI numbers (left) and gene numbers (right) per bin50 for 13 sections at time points ranging from E7.5 to E14.5 and 2 sections of HFD groups at E10.5 and E14.5. (e) Pairwise correlations of all sections. (f) Distributions of gene expression correlations between pairwise bin50 and scRNA data at time points ranging from E7.5 to E14.5.

Supplementary information, Fig. S2

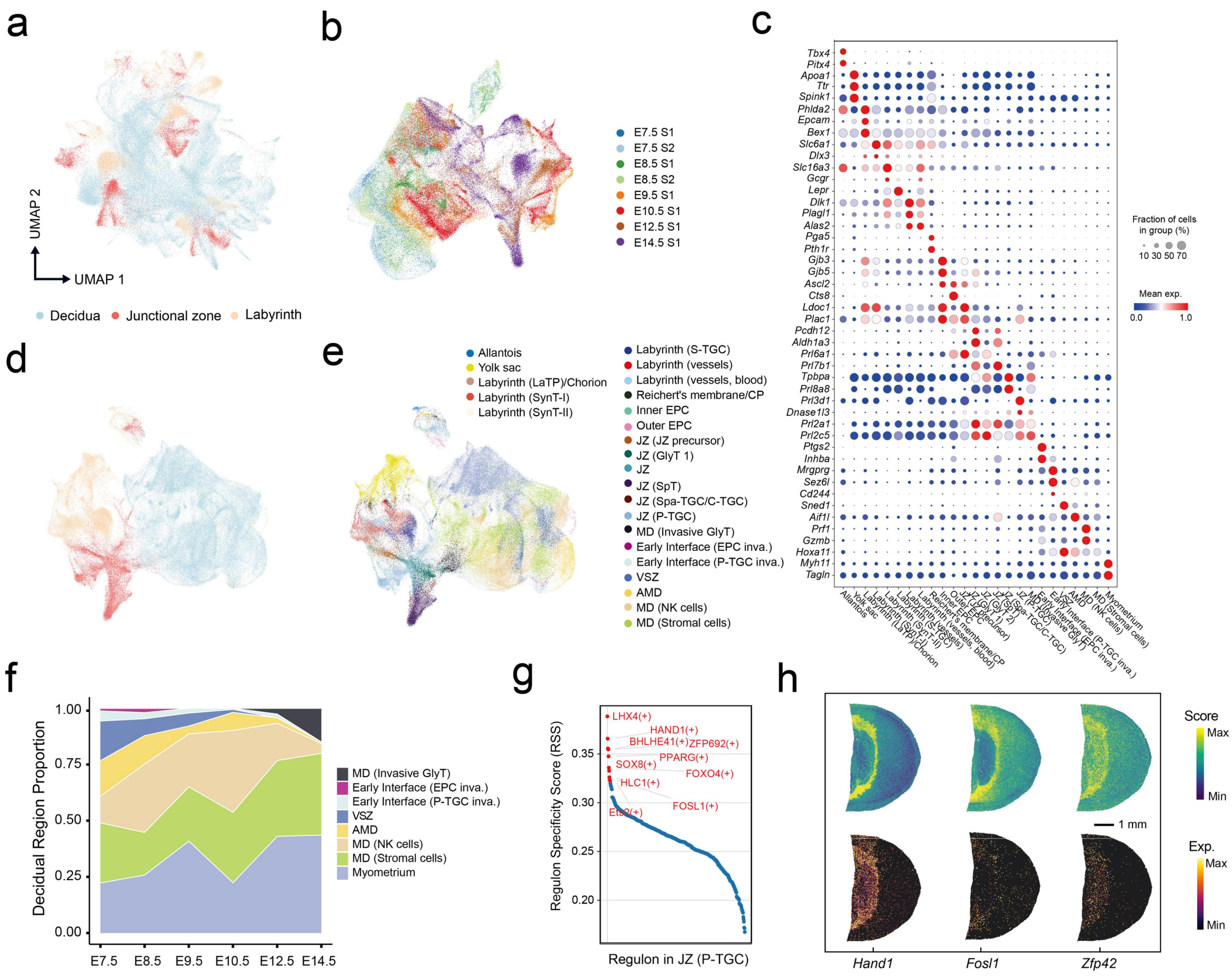

Supplementary information, Fig. S2: Spatiotemporal dynamics of placental clusters and regulon activities

UMAP plot showing bins from 8 sections before (a) and after the batch correction step (b), bins were colored by placental structures (a) and time points of sample collection (b) separately. (c) Averaged expression profiles for representative marker genes for each subregion. Sections of E7.5 S1, E7.5 S2, E8.5 S1, E8.5 S2, E9.5 S1, E10.5 S1, E12.5 S1 and E14.5 S1 were integrated for this analysis. (d) and (e) UMAP plot showing bins from all 13 uterine sections after batch correction, bins were colored by anatomical structures (d) and subregions (e). (f) Dynamic changes of the proportion for each decidual subregion captured at each developmental stage. (g) Regulon specificity distribution for cluster JZ (P-TGC) was shown with the top 10 TFs with the highest regulon activity score (RAS) score marked in red. (h) The spatial pattern of RAS and expression for *Hand1*, *Fosl1* and *Zfp42* at E9.5 were shown.

Supplementary information, Fig. S3

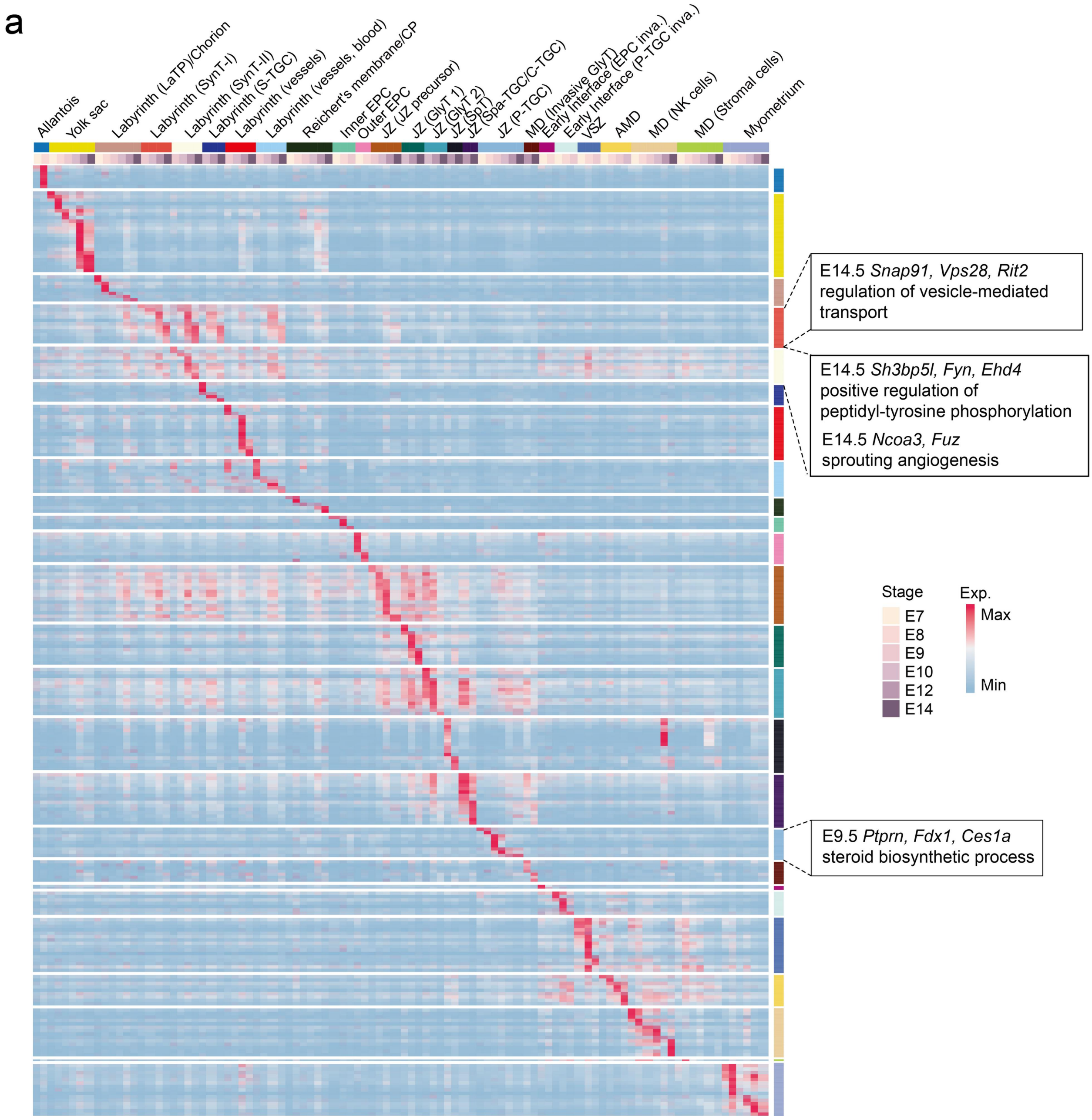

Supplementary information, Fig. S3: Spatiotemporal dynamics of placental gene expression

(a) Scaled SCTransform-normalized expression profiles of genes with regional specificity listed in Table S4, representative genes and related functional terms of the labyrinth (SynT-I), labyrinth (SynT-II) and JZ (P-TGC) subregions were displayed.

Supplementary information, Fig. S4

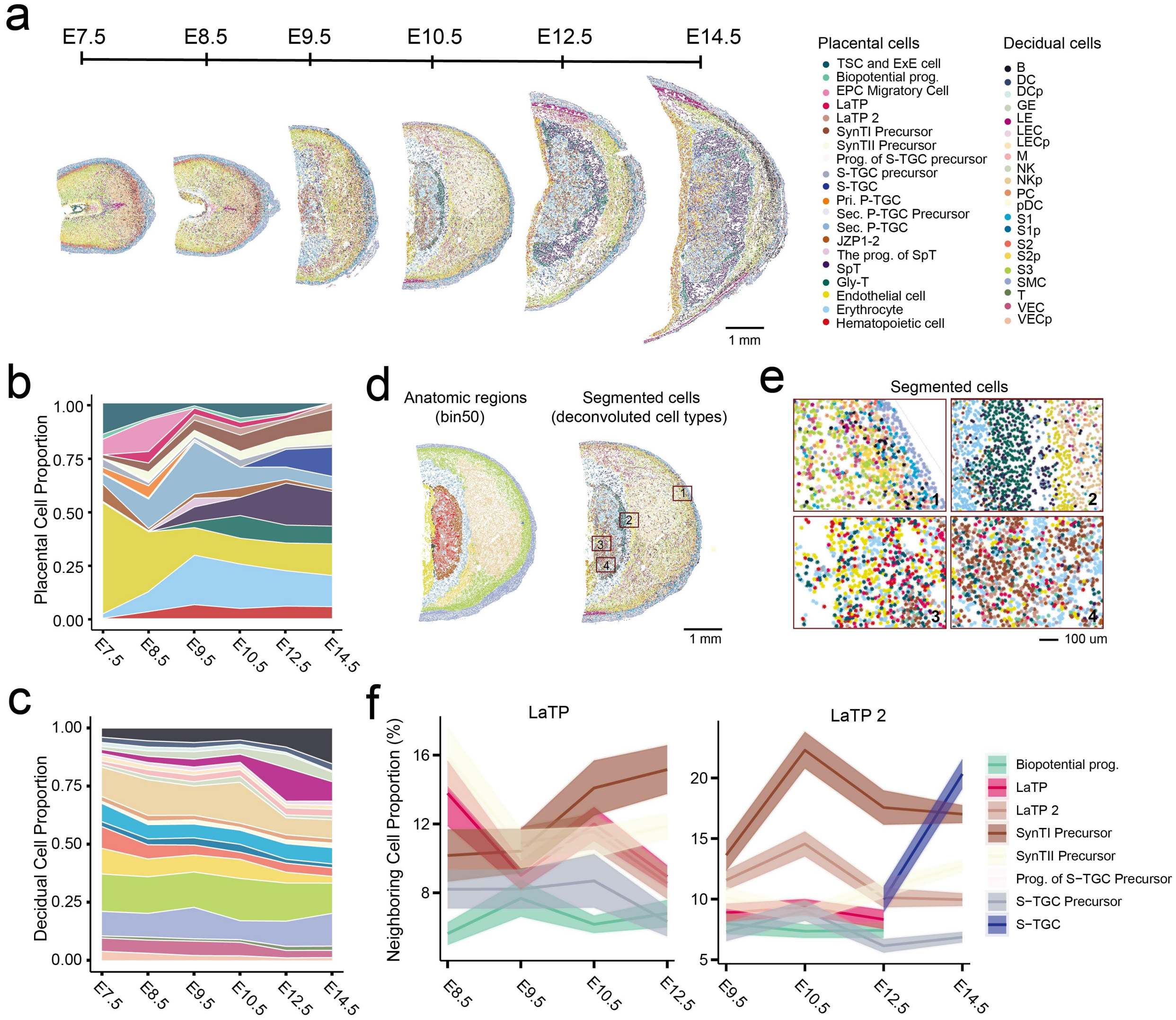

Supplementary information, Fig. S4: Temporal dynamics of cell types at whole placenta scale

(a) Spatial visualization of cell bins at time points ranging from E7.5 to E14.5. Cell bins are colored by deconvoluted cell types. (b) (c) Quantification of the proportion for placental cell types and decidual cell types captured at each developmental stage, all 13 uterine sections were used in this analysis. (d) Spatial visualization of the subregions and deconvoluted cell types of section E10.5. (e) Magnification images showing cell-type localization in the four regions squared in (d), the four regions focus on the decidualization process, the maternal-fetal interface, the SynT-I and SynT-II enriched regions, respectively. (f) Neighboring cell proportions of LaTP (left) and LaTP2 (right) (Endothelial cells, erythrocytes, hematopoietic cells excluded).

Supplementary information, Fig. S5

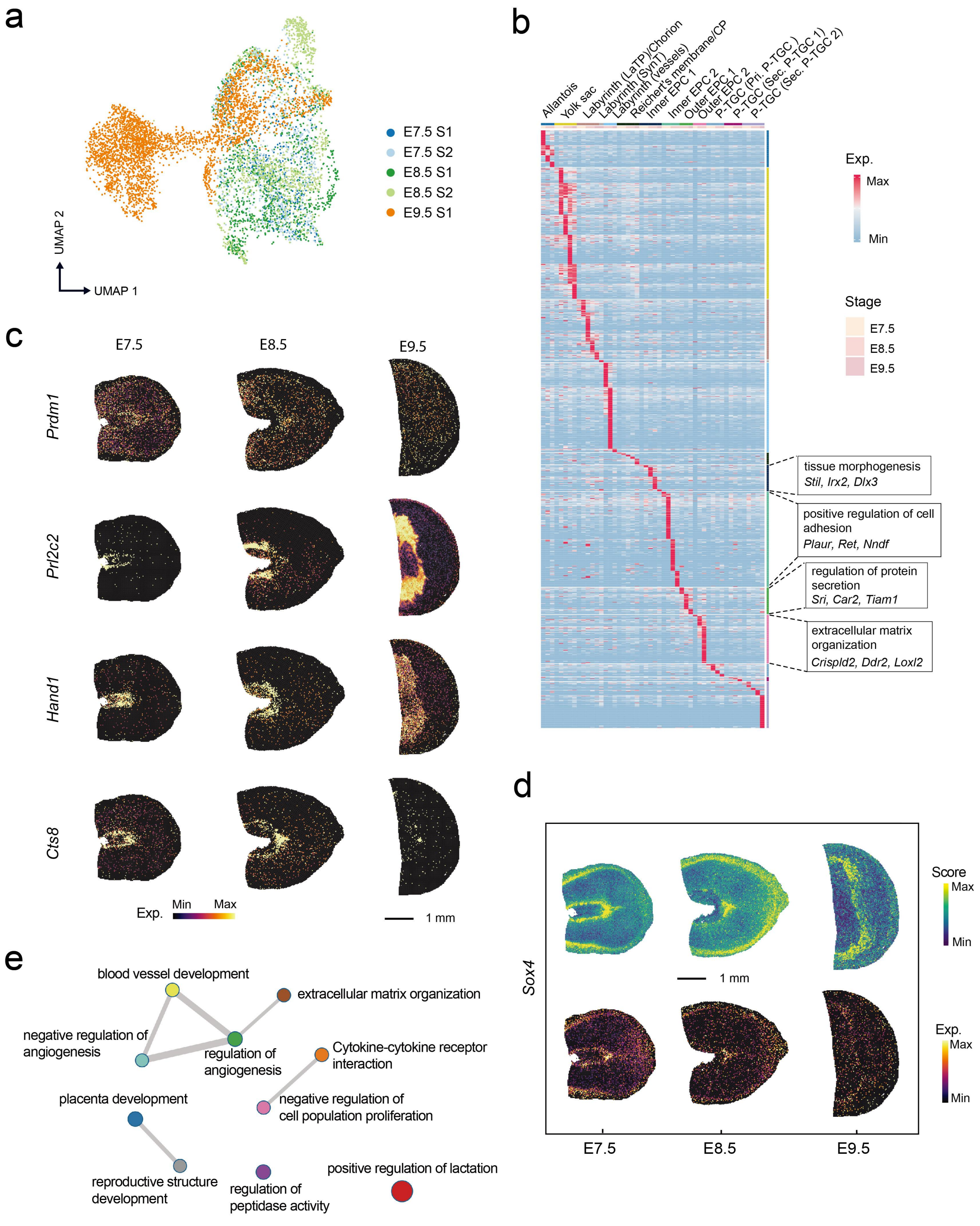

Supplementary information, Fig. S5: Functional gene regulations during early trophoblast development

(a) Trophoblast bins from five uterine sections from E7.5 to E9.5 were shown in UMAP after batch correction, with bins colored by each section. (b) Heatmap showing scaled SCTransform-normalized gene expression across different clusters among E7.5-E9.5 stages. Genes with spatiotemporal expression were included with the representative enriched biological process shown for the selected clusters. (c) Spatial expression profile of *Prdm1*, *Prl2c2*, *Hand1* and *Cts8* across E7.5 to E9.5 stages. (d) Spatial pattern of RAS and expression for *Sox4* across E7.5-E9.5 developmental stages. (e) Network of significantly enriched biological process terms (adjusted $p < 0.05$) for the outer EPC 2 subregion at E8.5 is visualized by Cytoscape.

Supplementary information, Fig. S6

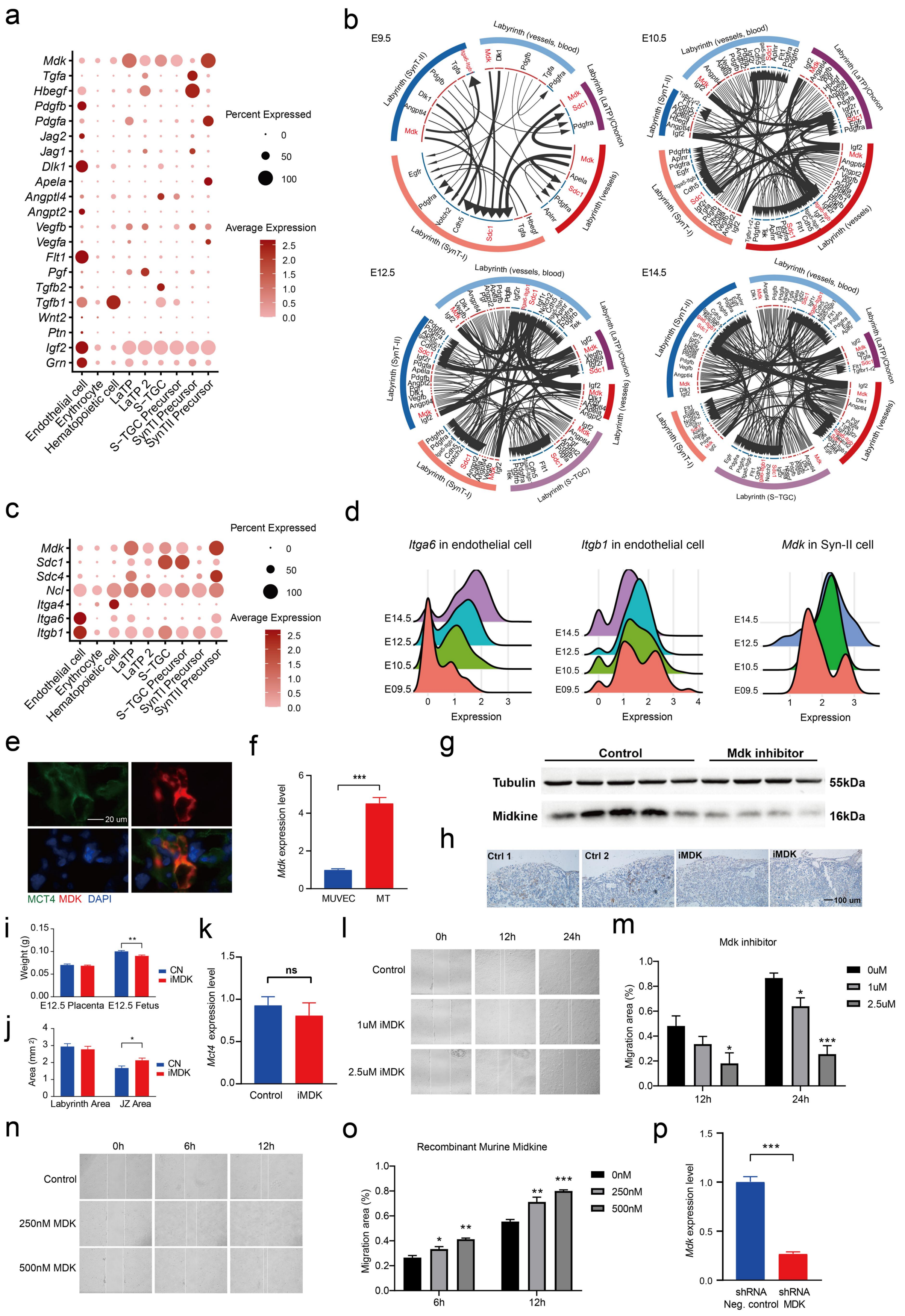

Supplementary information, Fig. S6: Spatial and single-cell resolved expression patterns of signaling events during labyrinth development

(a) Expression profiles of key hormones among different labyrinth cell types. (b) Snapshot of spatial interactions between different subregions captured at each stage from E9.5 to E14.5. (c) Expression of ligands and receptors in Midkine (MK) pathway among different labyrinth cell types. (d) Temporal dynamics of cell type-specific expression for *Itga6*, *Itgb1* and *Mdk*. (e) Immunofluorescence staining for the SynT-II marker (MCT4) and MDK indicating their colocalization in SynT-II in E12.5 mouse placenta sections (80x). (f) RT–qPCR analysis of *Mdk* expression in Mouse primary trophoblasts (MT) and Mouse primary umbilical vein endothelial cells (MUVEC). RT–qPCR data are normalized to the reference gene *Hprt*. (g) Western blotting results validating the inhibition effect of iMDK. (h) immunochemistry (IHC) staining showing the MDK expression with or without iMDK treatment. (i) Placental weight remains unchanged by iMDK treatment while fetal weight is decreased in the iMDK group compared to the control group (n=24 control, 41 iMDK litters). (j) Labyrinth area remains unchanged by iMDK treatment while JZ area is increased in iMDK group compared to the control group (n=8 control, 14 iMDK litters). (k) RT-qPCR results showing the expression of the SynT-II marker (*Mct4*). RT–qPCR data are normalized to the reference gene Hprt. The results are shown as the means ± SEMs. (l-o) The wound-healing assay demonstrating the capacity of migration in endothelial cells treated with iMDK (l-m)

and recombinant murine MDK (n-o). (p) RT–qPCR analysis of *Mdk* expression between shRNA-MDK-treated trophoblast cells and shRNA-Neg. control-treated trophoblast cells. The data are presented as the means ± SEMs. (*$p < 0.05$, **$p < 0.01$, ***$p < 0.001$ versus the control group, ns, not significant)

Supplementary information, Fig. S7

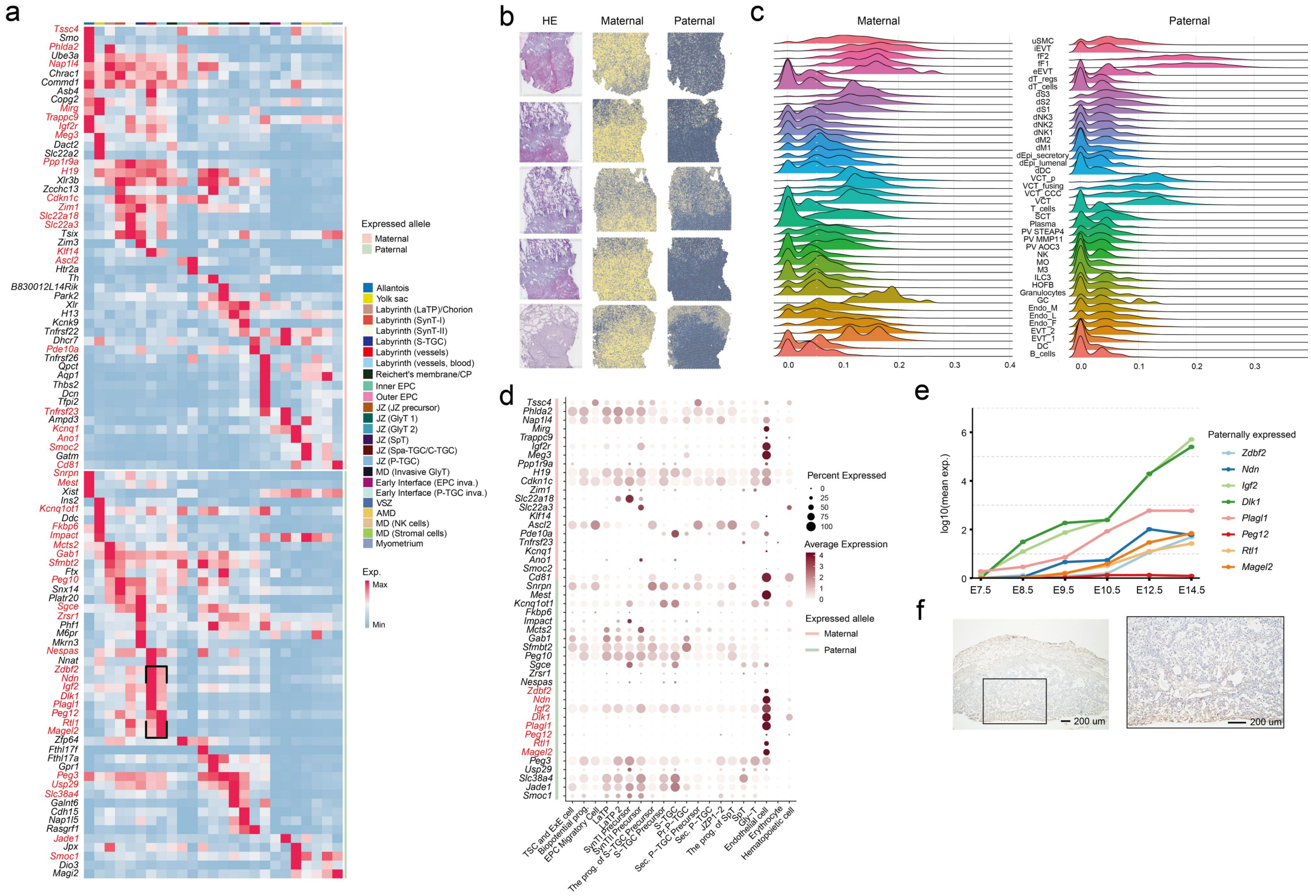

Supplementary information, Fig. S7: Expression profiling of imprinted genes.

(a) Spatial expression heatmap for all database-curated maternally and paternally expressed imprinted genes captured by spatial transcriptomics. Genes highlighted in red have had their allelic expression validated[3]. (b) Distribution of imprinted pattern in Visum spatial transcriptomics data of sections from donor P13 tissue[4]. Spot color indicates the Ucell score for orthologous gene sets that are validated as maternally and paternally expressed. (c) Cell type resolved distribution of Ucell score for imprinted gene sets across cell states in the human maternal-fetal interface[4]. (d) Cell type resolved expression profiles for allelically expressed imprinted genes. (e) Temporal dynamics of expression levels of selected paternally expressed genes in endothelial cells. (f) IHC staining showing the location of NDN expression in the chorionic plate and labyrinth. Right panel shows a magnificent view of the boxed field related to the left panel.

Supplementary information, Fig. S8

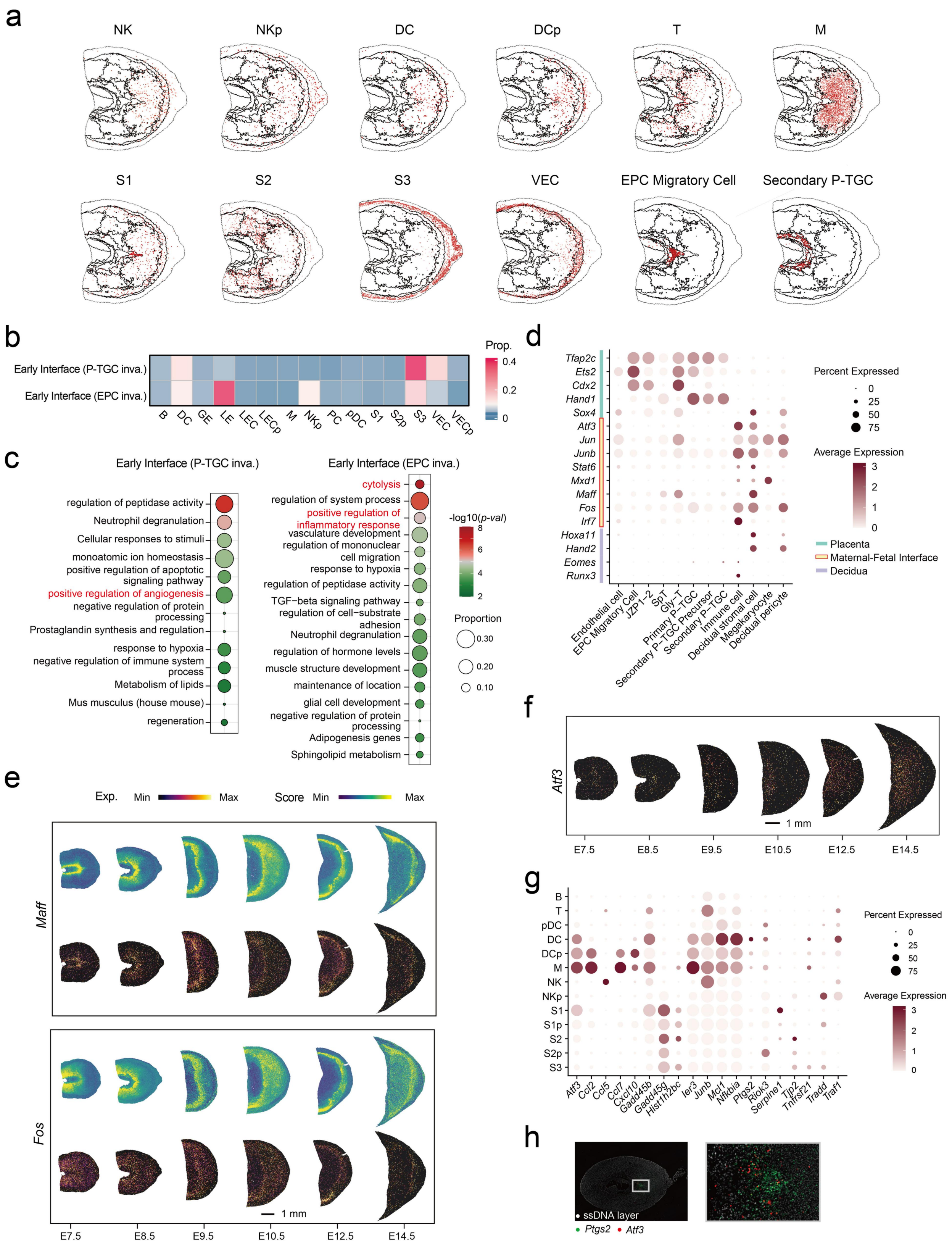

Supplementary information, Fig. S8: Transcriptomic regulation at the maternal-fetal interface

(a) The deconvolution results were displayed for each cell type separately for section E8.5 S1. (b) Cell proportion at early interface regions. Only cells with more than 1% are displayed. (c) Functional enrichment results based on the top 50 marker genes for early interface regions. (d) Gene expression for selected TFs among different cell types in published placental scRNA-seq data[1]. (e) Spatiotemporal dynamics of RAS and expression for *Maff* and *Fos* were shown. (f) Spatial expression profiles of *Atf3* across E7.5-E14.5. (g) Bubble plot showing expression profiles of *Atf3* targets in published decidual scRNA-seq data[2]. (h) Spatial visualization of the expression of *Ptgs2* and *Atf3* on ssDNA layer.

Supplementary information, Fig. S9

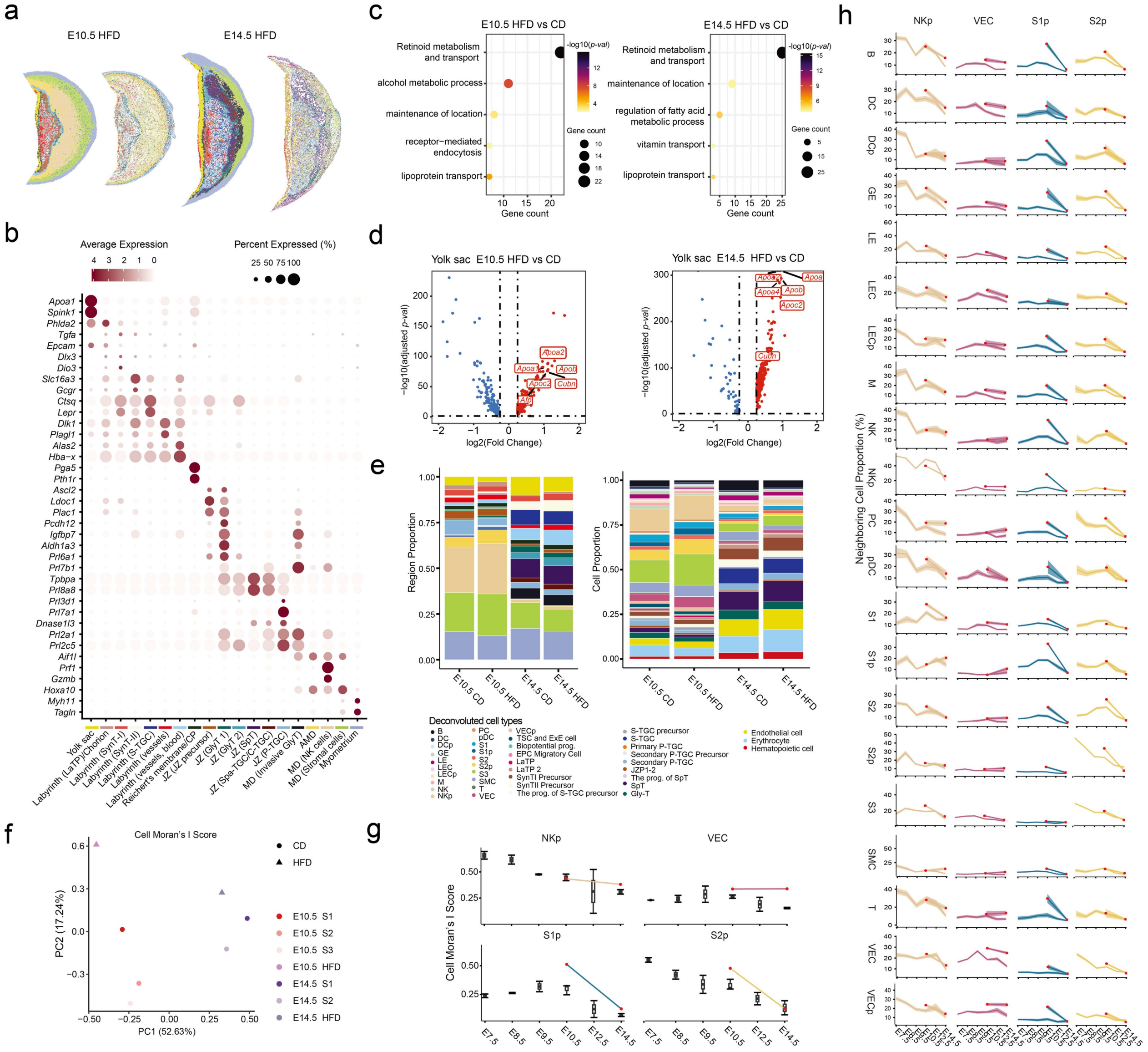

Supplementary information, Fig. S9: Characterization of HFD placenta sections.

(a) Bin50-level cluster mapping (left) and cell bin-level deconvolution landscape (right) of HFD placenta sections for E10.5 and E14.5. (b) Averaged expression profiles of representative markers for the E14.5 HFD section. Differentially expressed genes of the Yolk sac between CD and HFD groups were shown in (d) with representative enriched pathways visualized in (c). (e) Proportions of each subregion (left) and cell type (right) for both CD and HFD groups. (f) PCA plot of CD and HFD placenta sections for E10.5 and E14.5. Each point represents an individual section, with shapes indicating CD and HFD groups. (g) The trend of Moran's I score variation across developmental stages and groups is shown for selected cell types. Boxplots represent the CD groups, while red dots connected by lines indicate the HFD group. (h) The neighboring cell proportion of NKp, VEC, S1p and S2p cells for each decidual cell type was shown in each row. Ribbons represent developmental stages in CD groups, while red dots connected by lines indicate data from E10.5 HFD and E14.5 HFD.

Supplementary information, Fig. S10

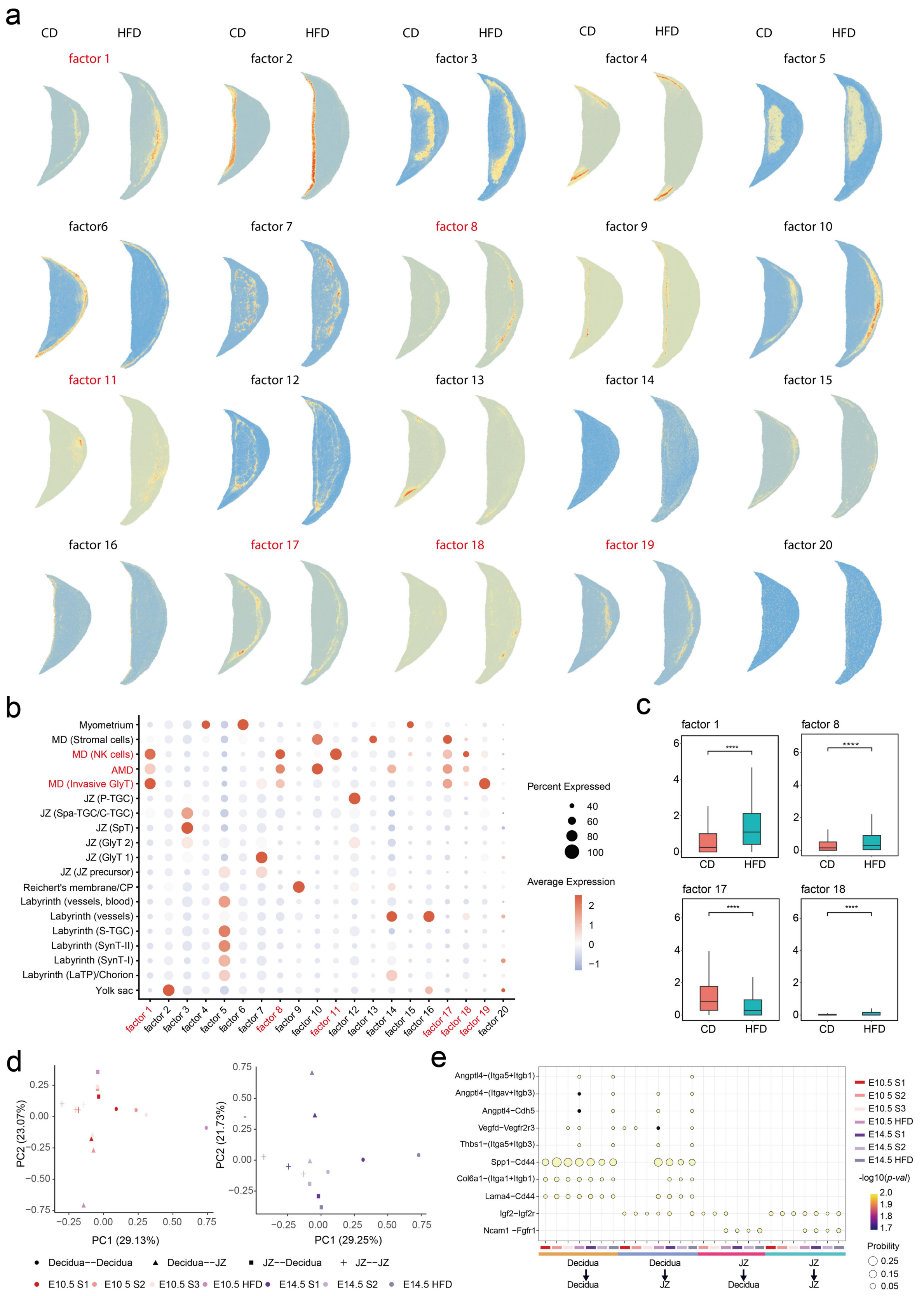

Supplementary information, Fig. S10: NNMF analysis of the HFD placenta sections.

Expression patterns of all 20 NNMF factors for E14.5 CD and HFD were displayed either in spatial space (a) or summarized for each subregion (b). (c) Comparison of NNMF weights for bins at the interface region between section E14.5 S1 and E14.5 HFD, ****$p < 0.0001$. (d) PCA plot on the interactome between JZ and decidua regions for E10.5 and E14.5 sections were shown separately. Each point represents the interactome between different regions in an individual section, with color indicating sections and shapes indicating group between different regions. (e) Interactions of selected ligand-receptor pairs between the decidua and JZ region across CD and HFD placenta sections for E10.5 and E14.5.

Supplementary information, Fig. S11

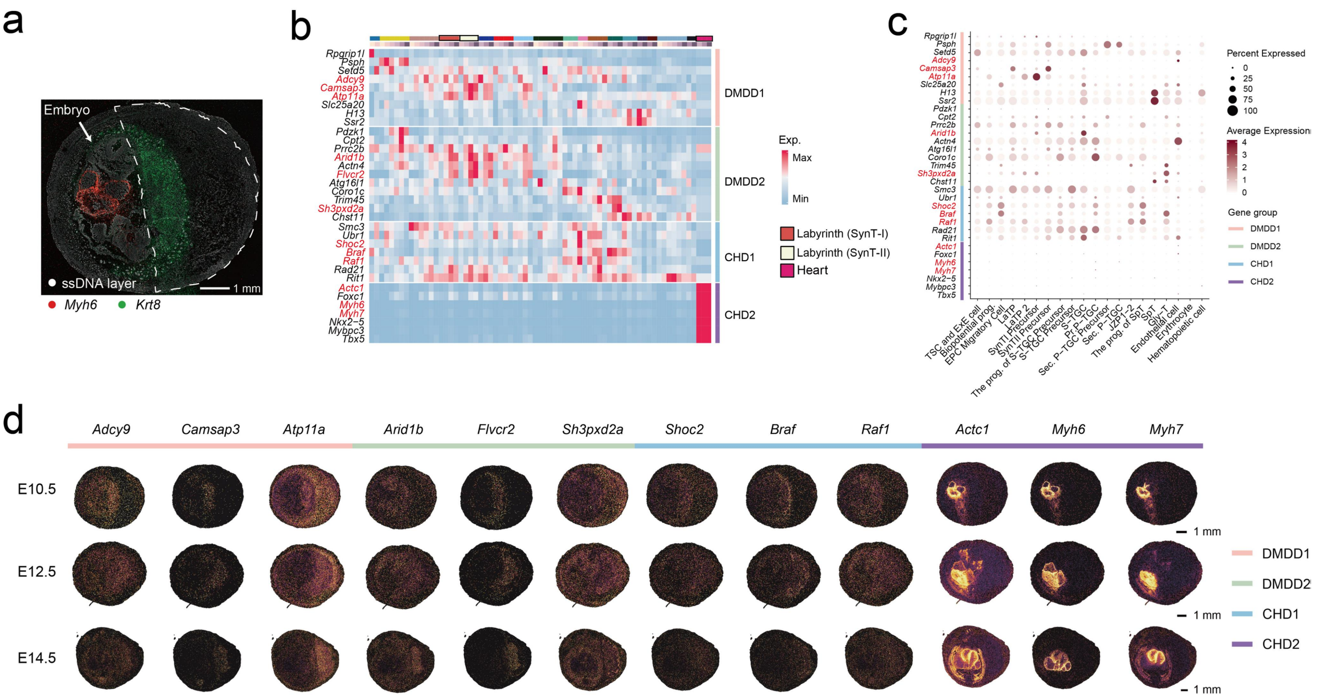

Supplementary information, Fig. S11: Spatial distribution of heart defect-associated genes in the placenta

(a) Spatial localization of *Myh6* and *Krt8* expression at E10.5 S1 section. (B, C) Overall expression profile for genes involved in abnormal heart development and/or placental defects across (b) spatial regions and (c) different cell types. (d) Spatial expression of selected genes in whole uterus sections at E10.5 S1, E12.5 S1 and E14.5 S1. Selected genes were highlighted in red in (b) and (c).

Captions Tables for S1 to S17

Table S1.

Marker genes for each subregion.

Table S2.

Abbreviations for each subregion.

Table S3.

Top 50 regulons with highest RSS for each subregion at E10.5.

Table S4.

Genes showing spatiotemporal specificity in Figure S3.

Table S5.

Metascape pathway analysis of genes expressed in syncytiotrophoblasts and polyploid trophoblast giant cells.

Table S6.

Genes showing spatiotemporal specificity in Figure 2.

Table S7.

Target genes of *Plagl1* at E14.5.

Table S8.

Genes significantly highly expressed at the early interface (P-TGC invasion) and early interface (EPC invasion).

Table S9.

Metascape pathway analysis of top 100 genes significantly highly expressed at the

early interface (P-TGC invasion) and early interface (EPC invasion).

Table S10.

Top50 target genes of *Atf3* at E8.5.

Table S11.

Metascape pathway analysis of *Atf3* top50 target genes at E8.5.

Table S12.

Top 50 genes for each NNMF factor.

Table S13.

Metascape pathway analysis of top genes from NNMF factor 1, 8, 17 and 18.

Table S14.

Target genes of *Irf7* at E14.5 HFD and CD.

Table S15.

Primer sequences for RT-qPCR.

Table S16.

Reported phenotypes for DMDD genes.

Table S17.

Curated phenotypes for CHD genes.

References

1 Jiang, X. et al. A differentiation roadmap of murine placentation at single-cell resolution. Cell Discov 9, 30, doi:10.1038/s41421-022-00513-z (2023).

2 He, J. P., Tian, Q., Zhu, Q. Y. & Liu, J. L. Single-cell analysis of mouse uterus at the invasion phase of embryo implantation. Cell Biosci 12, 13, doi:10.1186/s13578-022-00749-y (2022).

3 Gigante, S. et al. Using long-read sequencing to detect imprinted DNA methylation. Nucleic Acids Res 47, e46, doi:10.1093/nar/gkz107 (2019).

4 Arutyunyan, A. et al. Spatial multiomics map of trophoblast development in early pregnancy. Nature 616, 143-151, doi:10.1038/s41586-023-05869-0 (2023).
